# *SF3B1* mutation and *ATM* deletion codrive leukemogenesis via centromeric R-loop dysregulation

**DOI:** 10.1172/JCI163325

**Published:** 2023-09-01

**Authors:** Martina Cusan, Haifeng Shen, Bo Zhang, Aijun Liao, Lu Yang, Meiling Jin, Mike Fernandez, Prajish Iyer, Yiming Wu, Kevyn Hart, Catherine Gutierrez, Sara Nik, Shondra M. Pruett-Miller, Jeremy Stark, Esther A. Obeng, Teresa V. Bowman, Catherine J. Wu, Ren-Jang Lin, Lili Wang

**Affiliations:** 1 Department of Systems Biology, Beckman Research Institute of the City of Hope, Monrovia, California, USA.; 2 Department of Hematology, Union Hospital Tongji Medical College, Huazhong University of Science and Technology, Wuhan, China.; 3 Department of Hematology, Affiliated Shengjing Hospital of China Medical University, Shenyang, China.; 4 Irell and Manella Graduate School of Biological Sciences, Beckman Research Institute of the City of Hope, Duarte, California, USA.; 5 Department of Medical Oncology, Dana-Farber Cancer Institute, Boston, Massachusetts, USA.; 6 Gottesman Institute for Stem Cell Biology and Regenerative Medicine and; 7 Department of Developmental and Molecular Biology, Albert Einstein College of Medicine, New York, New York, USA.; 8 Department of Cell and Molecular Biology, St. Jude Children’s Research Hospital, Memphis, Tennessee, USA.; 9 Department of Cancer Genetics and Epigenetics, Beckman Research Institute of the City of Hope, Duarte, California, USA.; 10 Department of Oncology, St. Jude Children’s Research Hospital, Memphis, Tennessee, USA.; 11 Center for RNA Biology and Therapeutics, Beckman Research Institute of the City of Hope, Duarte, California, USA.

**Keywords:** Oncology, Cancer, DNA repair

## Abstract

RNA splicing factor *SF3B1* is recurrently mutated in various cancers, particularly in hematologic malignancies. We previously reported that coexpression of *Sf3b1* mutation and *Atm* deletion in B cells, but not either lesion alone, leads to the onset of chronic lymphocytic leukemia (CLL) with CLL cells harboring chromosome amplification. However, the exact role of *Sf3b1* mutation and *Atm* deletion in chromosomal instability (CIN) remains unclear. Here, we demonstrated that *SF3B1* mutation promotes centromeric R-loop (cen-R-loop) accumulation, leading to increased chromosome oscillation, impaired chromosome segregation, altered spindle architecture, and aneuploidy, which could be alleviated by removal of cen-R-loop and exaggerated by deletion of *ATM*. Aberrant splicing of key genes involved in R-loop processing underlay augmentation of cen-R-loop, as overexpression of the normal isoform, but not the altered form, mitigated mitotic stress in *SF3B1*-mutant cells. Our study identifies a critical role of splice variants in linking RNA splicing dysregulation and CIN and highlights cen-R-loop augmentation as a key mechanism for leukemogenesis.

## Graphical abstract

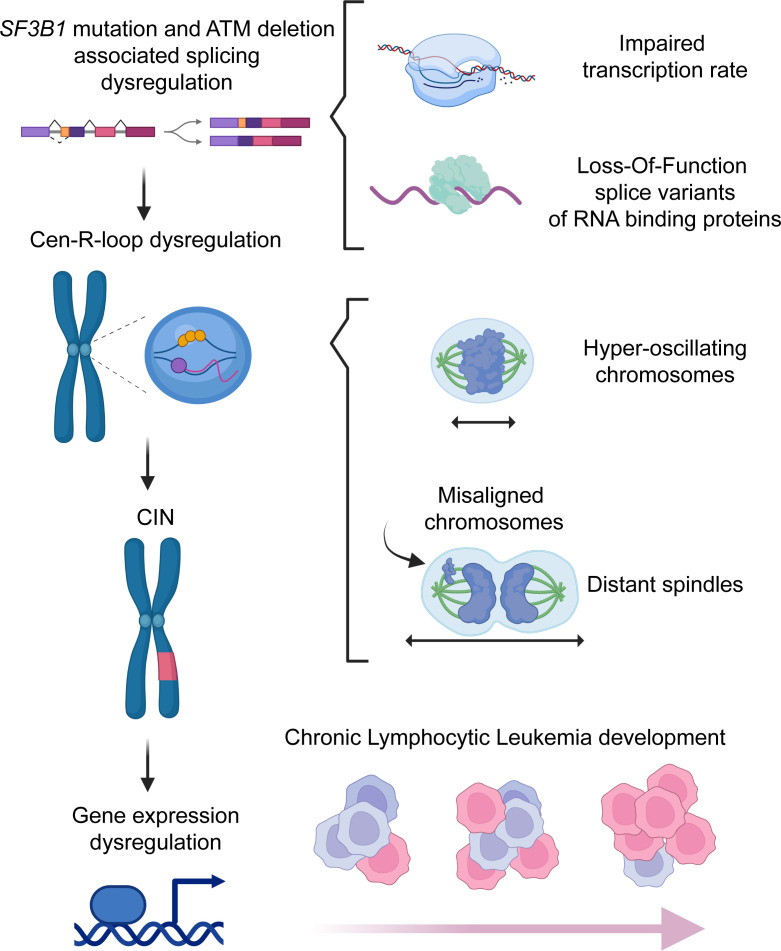

## Introduction

Cancer genome sequencing has identified high-frequency mutations of splicing factor *SF3B1* in hematologic malignancies such as myelodysplastic syndrome and chronic lymphocytic leukemia (CLL) (1–3). As one of the most common forms of adult leukemia in North America, CLL is characterized by clonal expansion of CD19^+^CD5^+^ cells in blood, bone marrow, and lymphoid organs (4). Mutations of *SF3B1* in CLL are clustered in hotspots (>50% at the K700E site) and co-occur with loss-of-function *ATM* mutations or deletion of chromosome 11q (minimal deleted region contains *ATM*) (1, 3). Our recent murine model confirmed that coexpression of *Sf3b1-*K700E with *Atm* deletion in B cells, but not either lesion alone, results in the onset of lowly penetrant CLL (5). These murine CLL cells exhibit RNA splicing dysregulation and genomic instability with recurrent amplification of chromosomes 15 and 17 (5), indicating that chromosomal instability (CIN) underlies CLL initiation; however, how these lesions contribute to CIN remains unclear.

CIN is a well-known hallmark of cancer that originates from inaccurate chromosome segregation ultimately leading to aneuploidy (6, 7). Faithful chromosome segregation relies on centromeres, a specialized chromosomal domain that serves as docking site for mitotic spindles during mitosis (8, 9). A recent study has demonstrated that formation of DNA:RNA hybrids (R-loops) at centromeres (cen-R-loops) is essential for CIN regulation (10). Splicing factor mutations in *SRSF2*, *U2AF1*, and *SF3B1* have been reported to promote global R-loop accumulation in myeloid cells (11, 12). As a well-known master regulator of DNA damage response (DDR), ATM can be activated by transcription-blocking lesions in an R-loop–dependent fashion, which further triggers spliceosome organization and promotes genome-wide alternative splicing (13), highlighting a reciprocal interaction between ATM kinase and RNA splicing. Whether *SF3B1* mutation and *ATM* deletion may impact CIN via cen-R-loop formation and RNA splicing dysregulation remains elusive.

Harnessing primary murine B cells and isogenic human cell lines with or without *SF3B1* mutation/*ATM* deletion, we investigated how these lesions, either alone or in combination, contribute to leukemogenesis via cen-R-loop augmentation.

## Results

### SF3B1 mutation triggers R-loop accumulation at centromeres.

**Figure 1:**
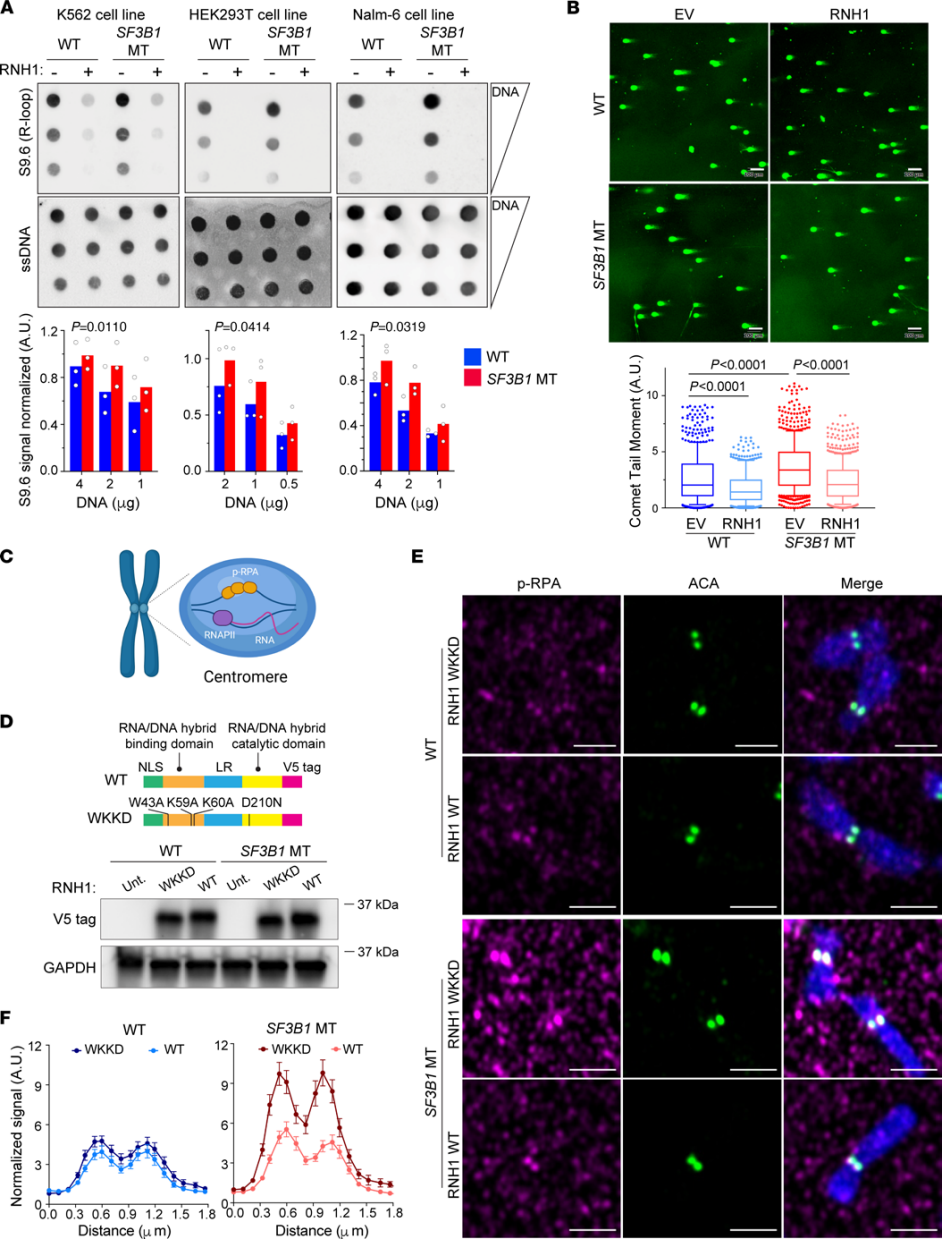
*SF3B1* mutation triggers cen-R-loop accumulation. (**A**) R-loop level quantified by dotblot assay with S9.6 antibody in K562, HEK293T, and Nalm-6 *SF3B1*-WT and -MT cells. Serial DNA dilutions starting from 4 mg (K562 and Nalm-6) or 2 mg (HEK293T). Single-strand DNA (ssDNA) blotting was used as loading control. Top: Representative image. Bottom: S9.6 signal quantification over ssDNA signal. Bar graphs represent mean; dots represent biological replicates. Two-way paired ANOVA test. (**B**) Representative images (top) of neutral comet assay for double-strand breaks in Nalm-6 *SF3B1*-WT and -MT cells with overexpression of either empty vector (EV) or RNaseH1 (RNH1) and relative comet tail moment (bottom) in 3 biological replicates. Scale bars: 100 μm. Total comets quantified range from 435 to 742 cells. Box plots show the median and 25th and 75th percentiles, with whiskers extending to minimum and maximum values. Two-tailed unpaired *t* test followed by Bonferroni’s post hoc test. (**C**) Cen-R-loops are recognized and coated by phospho–RPA S33 (p-RPA). RNAPII, RNA polymerase II. Created with BioRender (biorender.com). (**D**) Top: RNH1 WT and mutant vectors. NLS, nuclear localization signal; LR, linker region. Bottom: Detection of overexpression of RNH1 with V5 tag by immunoblot in Nalm-6 *SF3B1*-WT and -MT cells overexpressing either WT or WKKD RNH1 protein. GAPDH was used as loading control. (**E**) Representative images of cen-R-loops detected by p-RPA (red) and ACA (green) immunofluorescence. Scale bars: 2 μm. (**F**) Quantified centromeric p-RPA signal normalized to background signal near centromeres (see Methods). Graphs represent mean ± SEM. The number of chromosomes quantified ranges from 46 to 67. *SF3B1* MT overexpressing WKKD vs. WT RNH1, *P* = 0.0001, Wilcoxon’s paired test.

To explore whether *SF3B1* mutation promotes R-loop accumulation and DNA damage as a general mechanism across diverse cellular contexts, we measured R-loop level by dotblot assay in pre–B cell and myeloid cell lines (Nalm-6 and K562, respectively) with or without *SF3B1*-K700E mutation (14, 15). We also included a newly generated isogenic epithelial HEK293T cell line expressing *SF3B1*-K700E mutation at the endogenous locus for testing the generalization of *SF3B1* mutation and genomic instability (Supplemental Figure 1; supplemental material available online with this article; https://doi.org/10.1172/JCI163325DS1). R-loop level was elevated in *SF3B1*-mutant (MT) isogenic cell lines across diverse cellular contexts (Figure 1A), which was coupled with increased formation of DNA double-strand breaks (DSBs) measured by immunofluorescence (IF) and neutral comet assay (Figure 1B and Supplemental Figure 1F). Similarly, increased levels of DSBs and R-loops as quantified by S9.6 nuclear staining (Supplemental Figure 2, A and B) were confirmed in *Sf3b1*-MT murine B cells. To determine whether R-loop accumulation causes the DNA damage observed in *SF3B1*-MT cells, we overexpressed the R-loop–resolving enzyme RNaseH1 (RNH1, an enzyme that degrades the RNA moiety within DNA:RNA hybrid structures) in Nalm-6 and HEK293T isogenic cell lines and examined both DNA DSBs and R-loops. RNH1 overexpression not only dramatically reduced the S9.6 nuclear staining signal (Supplemental Figure 2, C and D) but also alleviated DNA DSB formation in *SF3B1*-MT cells (Figure 1B and Supplemental Figure 2, C and E) in both cellular contexts, indicating that *SF3B1* mutation–associated R-loop augmentation is sufficient to induce DNA DSBs.

R-loops can be harmful, but they are essential for proper chromosome segregation during mitosis (10, 16). To investigate whether *SF3B1* mutation also promotes cen-R-loop formation, we measured serine 33–phosphorylated replication protein A (phospho-RPA [p-RPA]) — an established marker for cen-R-loops (10) — localized at centromeres by IF staining with anti–p-RPA and anti-centromere antibodies (ACA) on metaphase chromosome spreads (Figure 1C). Centromeric p-RPA signal was higher in *SF3B1*-MT compared with WT cells in all isogenic cell lines tested (*P* < 0.001; Supplemental Figure 3A). Moreover, we generated *SF3B1*-WT and -MT Nalm-6 cell lines overexpressing either WT RNH1 or a mutant form (WKKD RNH1; residues mutated: W43A, K59A, K60A) that is unable to bind or resolve R-loops (17) (Figure 1D). Overexpression of WT RNH1 greatly reduced centromere p-RPA coating compared with WKKD RNH1 in *SF3B1*-MT cells (Figure 1, E and F). These data strongly suggest that *SF3B1* mutation not only triggers unscheduled R-loop accumulation but also alters physiologic DNA:RNA hybrid formation/resolution over centromeres.

### Dysregulation of cen-R-loops leads to CIN.

**Figure 2:**
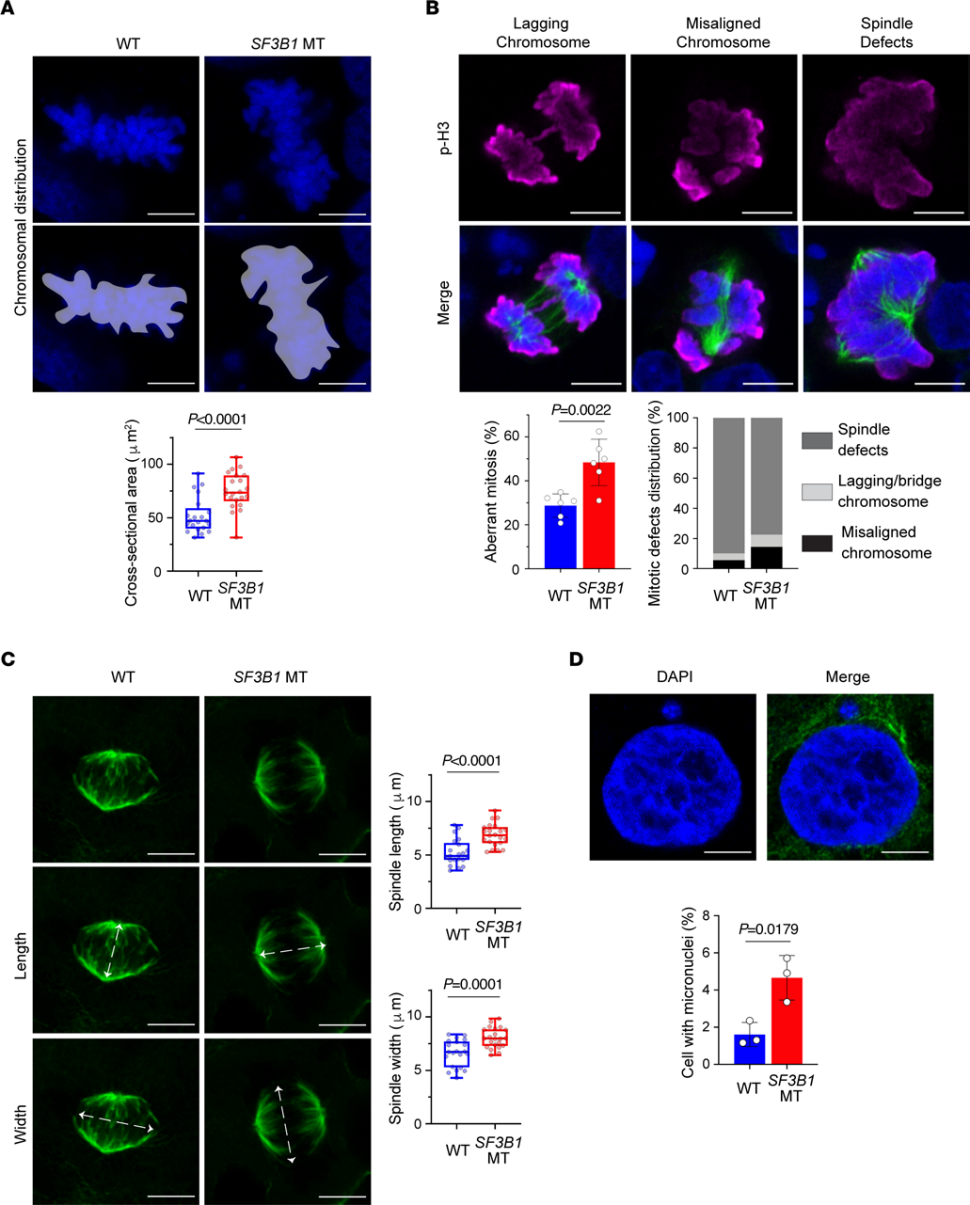
*SF3B1*-mutant cells have mitotic stress, spindle structure defects, and micronuclei. (**A**) Top: Representative confocal maximum intensity projections of entire *Z*-stack images for measurement of chromosome distribution and alignment during metaphase. Scale bars: 5 μm. Purple areas indicate the area measured. Bottom: Quantification of chromosome area above. (**B**) Top: Representative images of mitotic cells with lagging chromosomes and chromosomes bridges, misaligned chromosomes, and multipolar spindles. Mitotic cells marked with H3–serine 10 (p-H3) antibody (magenta); spindles marked with α-tubulin antibody (green); nuclei marked with DAPI (blue). Scale bars: 5 μm. Bottom: Quantification of aberrant mitosis frequency, expressed as percentage of total mitosis encountered, and distribution of mitotic defects expressed as percentage of total aberrant mitotic cells. (**C**) Left: Representative maximum intensity projections of mitotic spindle architecture of cells in metaphase. Arrows indicate definition of length (middle panel) and width (bottom panel). Green, α-tubulin. Scale bars: 5 μm. Right: Relative spindle length and width quantification. (**D**) Top: Representative image of cell with micronuclei. Blue, nuclei (DAPI); green, α-tubulin. Scale bars: 5 μm. Bottom: Quantification of frequency of micronuclei. Data are expressed as percentage of total cells. All panels show data in HEK293T *SF3B1*-WT and -MT cells. Box plots show the median and 25th and 75th percentiles, with whiskers extending to minimum and maximum values. Bar plots represent mean ± SD. Each dot represents a biological replicate. Two-tailed unpaired *t* test.

**Figure 3:**
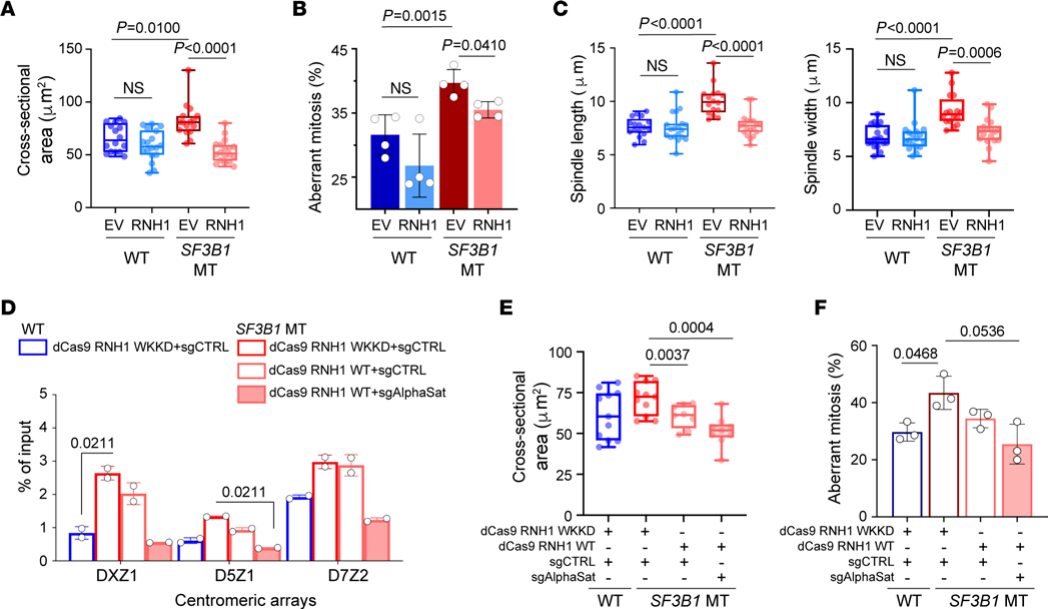
*SF3B1* mutation–associated cen-R-loop dysregulation leads to aneuploidy. (**A**–**C**) Analysis of 2-dimensional cross-sectional area of the entire body of chromosomes (**A**) and aberrant mitosis frequency (**B**) and spindle length and width (**C**) in HEK293T *SF3B1* WT and MT overexpressing either empty vector (EV) or WT RNH1. (**D**) DRIP-qPCR in HEK293T *SF3B1* WT and MT overexpressing either dCas9–GFP–RNaseH1 WKKD (WKKD RNH1) or dCas9–GFP–RNaseH1 WT (WT RNH1) in combination with either sgRNA guide control (sgCTRL) or sgRNA targeting α-satellite centromeric repeats (sgAlphaSat). Centromeric arrays: The chromosome is specified by the number following the “D”; and the array is specified by the number following the “Z.” Two-way ANOVA test. (**E** and **F**) Analysis of 2-dimensional cross-sectional area of the entire body of chromosomes (**E**) and aberrant mitosis frequency (**F**) in cells from **D**. Box plots show the median and 25th and 75th percentiles, with whiskers extending to minimum and maximum values. Bar graphs represent mean ± SD. Each dot represents a biological replicate. Two-tailed unpaired *t* test followed by Bonferroni’s post hoc test, except in **D**.

To investigate the consequences of disrupted cen-R-loop homeostasis associated with *SF3B1* mutation, we examined several features that potentially influence CIN. We first determined the magnitude of chromosome oscillations during metaphase by measuring the total body of chromosome-occupied area (Figure 2A). A greater range of chromosomal oscillations in *SF3B1*-MT compared with WT cells was observed in both HEK293T and Nalm-6 cell lines (Figure 2A and Supplemental Figure 3, B and C), suggesting that an increased level of cen-R-loops impacts centromere–mitotic spindle dynamics. As mitotic spindles ensure proper alignment and distribution of chromosomes during mitosis, we quantified mitotic defects in *SF3B1*-WT and -MT isogenic cell lines using IF against phospho–serine 10 histone H3 (p-H3Ser10, a mitotic cell marker) and α-tubulin (a mitotic spindle marker) (18). Aberrant mitosis was defined as cells presenting lagging chromosomes, multipolar spindles, and misaligned chromosomes (Figure 2B). Compared with WT, we observed a higher percentage of aberrations in *SF3B1*-MT cells, indicating that the mutation significantly affected the mitotic process (HEK293 *SF3B1* WT ~28% vs. MT ~52%, Nalm-6 *SF3B1* WT ~30% vs. MT ~47%, K562 *SF3B1* WT ~32% vs. MT ~45%) (Figure 2B and Supplemental Figure 3, D and E). Consistent with the hyper-oscillating chromosomes, *SF3B1* MT induced an increased fraction of cells harboring chromosome misalignment (Figure 2B and Supplemental Figure 3E). When the mitotic spindle architecture was analyzed, *SF3B1*-MT cells presented wider and more distant mitotic spindles compared with WT cells (*P* < 0.001; Figure 2C and Supplemental Figure 3, B and F). As a result of severely misaligned chromosomes traversing longer distances during anaphase, we observed an increased frequency of micronuclei in *SF3B1*-MT cells (*P* = 0.0179; Figure 2D). Moreover, RNH1 overexpression reduced excessive chromosomal oscillation during metaphase (*P* < 0.0001; Figure 3A and Supplemental Figure 3, G and H), lowered aberrant mitosis frequency (*P* = 0.041; Figure 3B and Supplemental Figure 3I), and decreased mitotic spindle length and width (*P* = 0.0006; Figure 3C and Supplemental Figure 3, G and L) in *SF3B1*-MT cells. These results suggest that dysregulation of cen-R-loops leads to CIN.

To determine whether mitotic stress observed in *SF3B1*-MT cells is due to R-loop accumulation at centromere but not elsewhere, we used a dCas9-eGFP-RNH1 fusion protein coupled with single-guide RNA (sgRNA) targeting the α-satellite repetitive sequences (sgAlphaSat) to modulate cen-R-loops (19, 20) (Figure 3D and Supplemental Figure 4A). As a control, we included dCas9–eGFP–RNH1 WKKD form, which could bind to the targeted region via sgRNA but does not resolve DNA:RNA hybrids, leading to site-specific promotion of R-loops. Consistent with a previous report (19), dCas9-eGFP-RNH1 displayed nuclear and nucleolar localization in the presence of sgRNA control (sgCTRL) but changed to a nuclear distribution with puncta in the presence of sgAlphaSat (Supplemental Figure 4A). We first confirmed that the dCas9-eGFP-RNH1 system can proficiently modulate R-loop formation in a site-specific manner using sgRNA targeting the *Actin* locus (sgActin). Our DNA:RNA hybrid immunoprecipitation–quantitative (DRIP-quantitative) PCR (qPCR) revealed that R-loops around a 5′ pausing site (known to form R-loops) were upregulated in dCas9–eGFP–RNH1 WKKD cells but downregulated in dCas9-eGFP-RNH1 cells with sgActin compared with sgCTRL (Supplemental Figure 4B). Moreover, sgRNA targeting *Actin* intron 1 region, a negative locus for R-loop formation, in dCas9–eGFP–RNH1 WKKD cells led to upregulated R-loop formation over this region but caused no changes in dCas9-eGFP-RNH1 cells, validating the specificity of this system. We then examined the modulation of cen-R-loops with sgAlphaSat. Cen-R-loops were efficiently downregulated over several chromosome centromeric regions in dCas9-eGFP-RNH1 cells with sgAlphaSat compared with sgCTRL (Figure 3D). As a result, disruption of cen-R-loop accumulation alleviated chromosomal oscillations and defective mitosis more efficiently than targeting to control region in HEK293T *SF3B1*-MT cells, although with subtle impacts on spindle architecture (Figure 3, E and F, and Supplemental Figure 4C). Our results collectively demonstrate that cen-R-loop accumulation associated with *SF3B1* mutation reduces chromosomal stability and alignment during mitosis, eventually leading to CIN and an aberrant chromosomal burden.

### R-loop accumulation loci do not coincide with genes undergoing aberrant splicing.

**Figure 4:**
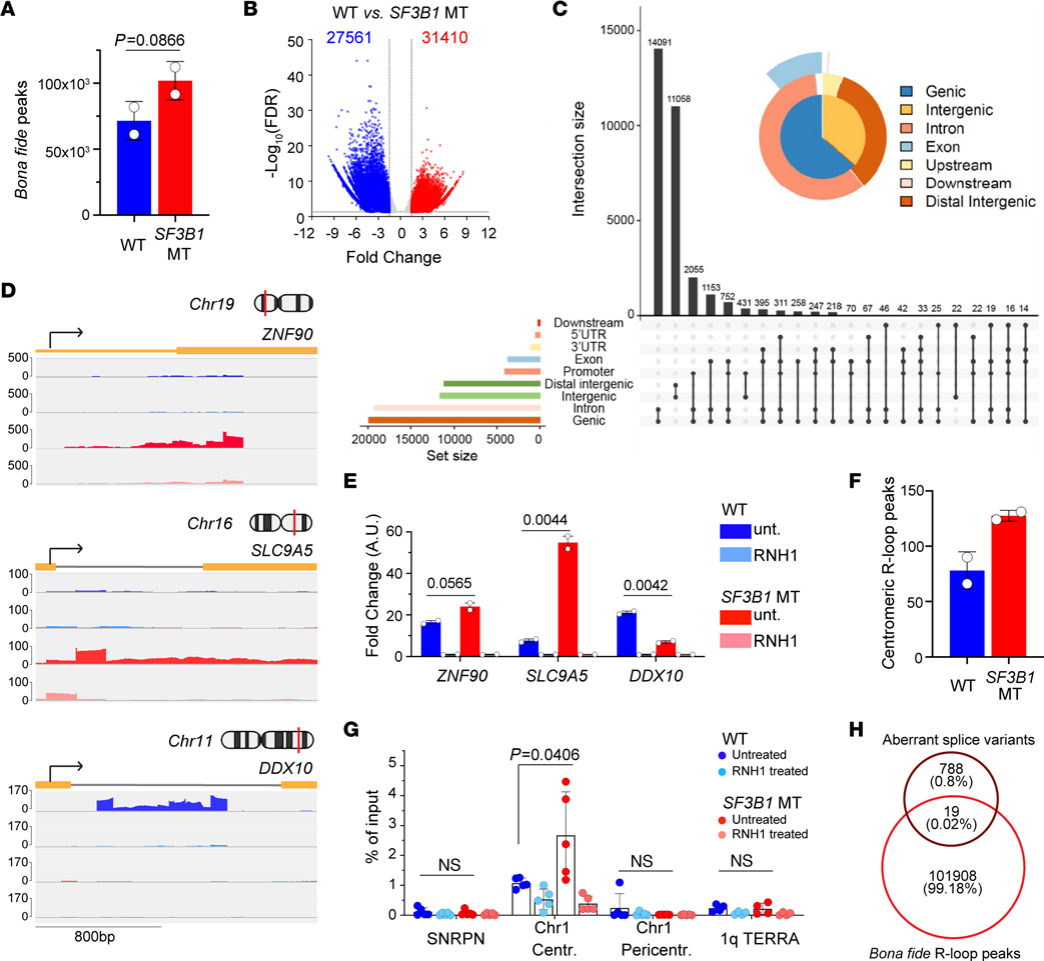
*SF3B1* mutation–associated R-loops have minimal overlapping with splice variants. (**A**) Quantification of genome-wide bona fide R-loops in Nalm-6 WT and *SF3B1* MT detected by DRIP-seq. (**B**) Volcano plot of differential bona fide R-loops between *SF3B1*-MT and -WT cells. Significant differential peaks cutoff as FDR < 0.05 and fold change > 1.5. (**C**) Genomic distribution of differential upregulated bona fide R-loops associated with *SF3B1* mutation with UpSet and PieChart plots. Intersection size indicates the number of R-loops. The black dots connected with lines represent overlapped R-loops. (**D**) Integrative Genomics Viewer (IGV) of R-loops profiled by DRIP-seq over indicated upregulated and downregulated genes. (**E**) Validation of differential R-loop peaks in **D** by DRIP-qPCR assay. RNH1 treatment is included as background control. Graphs represent qPCR results of biological duplicates; fold change over paired RNH1 treatment is presented as mean ± SEM; 2-tailed unpaired *t* test. (**F**) Quantification of centromeric bona fide R-loops detected by DRIP-seq. (**G**) *SF3B1* mutation–associated cen-R-loops validated using DRIP-qPCR in mitotic cells with and without *SF3B1* mutation. Chromosome 1 (Chr1) centromere, pericentromere, and telomeric 1q TERRA regions tested for R-loop accumulation. *SNRPN* was used as negative control. RNH1-treated samples were used as an R-loop background control. Graphs represent qPCR results expressed as percentage of input mean ± SEM. Dots represent technical replicates of 2 biological replicates. (**H**) Venn diagram demonstrates overlap between Nalm-6 *SF3B1* MT–associated alternative splice variants and bona fide R-loop peaks in WT (blue) and MT (red) *SF3B1*.

To determine whether *SF3B1* mutation–associated RNA splicing dysregulation may directly affect R-loop accumulation, we performed DRIP using the S9.6 antibody followed by sequencing (DRIP-seq) to localize R-loops in a genome-wide fashion (21) (see Methods; and Supplemental Figure 5, A–C). In this manner, we identified bona fide R-loop peaks by comparing paired samples with and without RNH1 treatment and detected an average of 71,590 and 101,928 total bona fide peaks in *SF3B1-*WT and -MT cells, respectively (Figure 4A). The overall distribution of R-loops across the genome in *SF3B1-*WT and -MT cells was similar (Supplemental Figure 5, D and E), but with greater accumulation at promoter regions (*P* < 0.0001, 2-way ANOVA) and exons (*P* = 0.0002, 2-way ANOVA) in MT cells (Supplemental Figure 5E).

To further identify genomic regions differentially perturbed by *SF3B1* mutation, we measured the net gain of R-loops in human *SF3B1*-MT versus WT cells (fold change = 1.5, FDR < 0.05). We identified a total of 27,561 downregulated and 31,412 upregulated bona fide peaks in *SF3B1*-MT compared with WT cells (Figure 4B). Differential peaks were mostly located over introns, intergenic regions, and promoters; to a lesser extent, they were also noted over exons (Figure 4C). Three differential peaks with greater difference between WT and MT cells were then confirmed by Integrative Genomics Viewer visualization and DRIP-qPCR (Figure 4, D and E).

DRIP-seq R-loop profiling over chromosomes confirmed a greater accumulation of peaks at the centromeric regions of different chromosomes in *SF3B1-*MT compared with WT cells (Figure 4F and Supplemental Figure 6). In the example of chromosome 1, we experimentally validated an accumulation of R-loops over centromeric but not pericentromeric nor telomeric regions by DRIP-qPCR (Figure 4G) in *SF3B1*-MT cells with well-defined primers (Supplemental Table 1).

To examine the relationship between splicing dysregulation and R-loop accumulation, we overlapped *SF3B1* mutation–associated alternative splice variants (Supplemental Table 2) and the genomic locations of R-loop peaks. This analysis revealed minimal overlap of the identified splice variants with bona fide R-loop peaks (only 19 splice variants, or 0.02% of total peaks) (Figure 4H). Furthermore, we quantified *SF3B1* mutation–associated splicing changes by qPCR in a set of transcripts selected based on the highest extent of splicing changes (*DYNLL1*, *MTERF2*, *DLST*, *TMEM14C*, *HLTF*, *TGFBR1*, *EHMT1*, *TRIM37*, *ORAI2*) and found them to be only subtly affected by RNH1 overexpression (Supplemental Figure 7A). These results indicated that *SF3B1* mutation*–*associated R-loop accumulation is unlikely to be caused by a direct interaction of aberrantly spliced RNA molecules with neighboring DNA. Altogether, these observations led us to consider whether a general mechanism, such as regulation of transcription rate or the products of aberrantly spliced transcripts, may directly influence *SF3B1* mutation–associated R-loop accumulation.

### Aberrant splicing of RNA-binding proteins causes R-loop and DNA damage accumulation.

Multiple lines of evidence have tightly linked formation of functional spliceosome to mRNA biogenesis. Mutation in *SRSF2* has been demonstrated to negatively regulate transcription rate by causing RNA polymerase II (RNAPII) pausing to contribute to R-loop accumulation (11). The U2 small nuclear ribonucleoprotein has been shown to be required for efficient RNAPII pause release and to influence elongation velocity, impacting transcription (22). *SF3B1* mutation has been recently reported to induce RNAPII transcription elongation defect through impaired assembly of early spliceosome complexes (23). Accordingly, quantifying nascent RNA synthesis using nuclear incorporation of the modified RNA precursor 5-ethynyluridine (EU) via IF, we detected decreased rates of RNA synthesis in *Sf3b1*-MT murine splenic B cells and Nalm-6 cells (Supplemental Figure 7, B and C) compared with WT cells.

We then sought to further explore whether *SF3B1* mutation likewise triggers R-loop accumulation via alternative splicing of genes involved in R-loop biogenesis. Given that RNA splicing regulation often acts in a cell context–dependent manner (24), we first identified 96 commonly shared splicing events associated with *SF3B1* mutation using RNA sequencing (RNA-Seq) data derived from our panel of isogenic cell lines (Supplemental Figure 8A, 3.3% of total splice events considered; and Supplemental Table 2) (14). Gene Ontology analysis of these shared splice variants revealed mRNA decay, DDR, cell cycle, and NF-κB signaling as key pathways commonly affected by *SF3B1* mutation (Supplemental Figure 8B). By extension, we wondered whether *SF3B1* mutation could influence CIN through one or more of these pathways.

**Figure 5:**
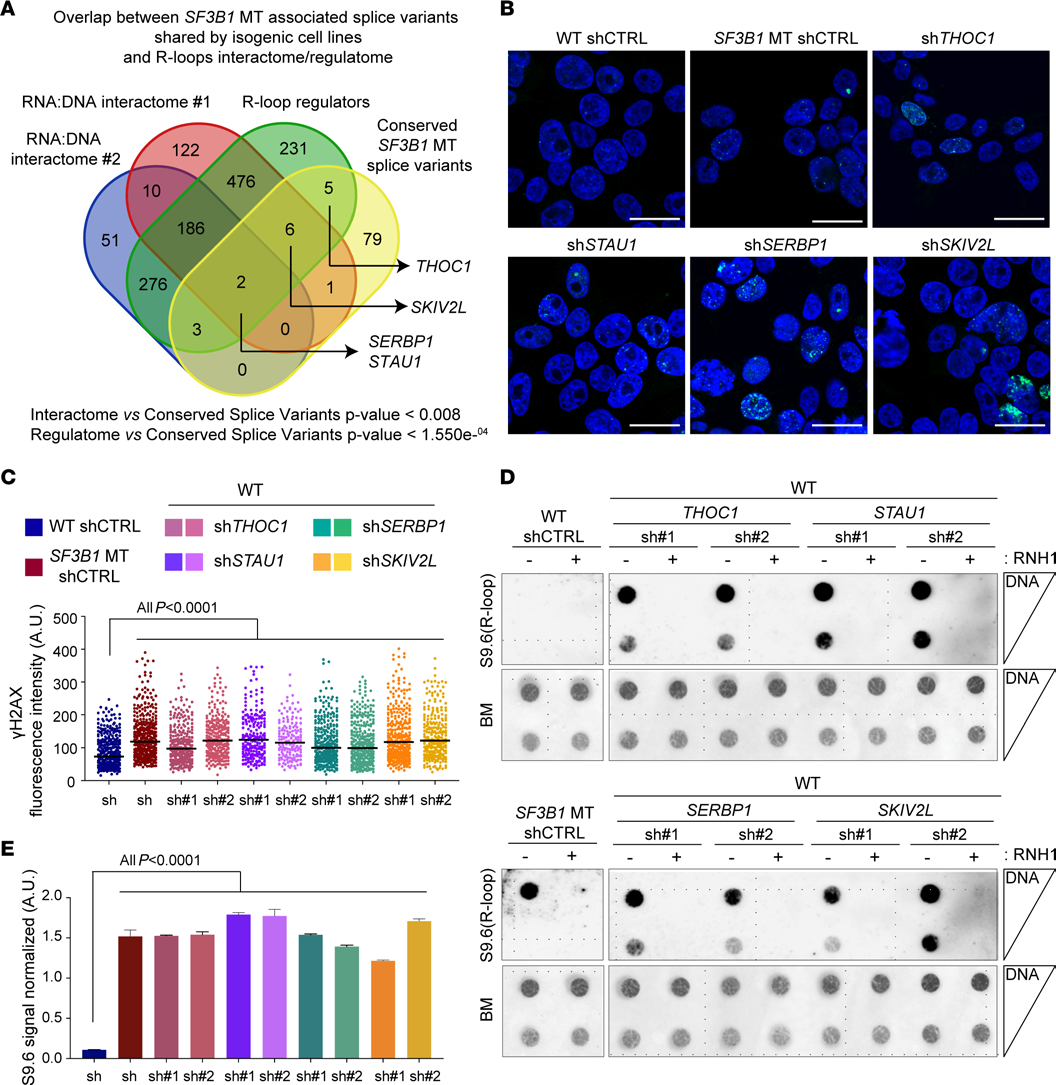
R-loop accumulation is induced by *SF3B1* mutation–associated loss-of-function alternative splice variants. (**A**) Venn diagram of overlapped conserved splice variants among *SF3B1*-MT isogenic cell lines (HEK293T, K562, and Nalm-6; Supplemental Table 1); DNA:RNA hybrid interactome in human cell studies: interactome #1 (26), interactome #2 (21, 28), and R-loop regulatory proteins data set (27). Hypergeometric distribution test. (**B**–**E**) Representative images of γH2AX foci immunofluorescence staining (**B**), R-loops detected by dotblot analysis (**D**), and relative quantification (**C** and **E**) in HEK293T cells with silencing of *THOC1*, *STAU1*, *SERBP1*, and *SKIV2L* genes. γH2AX foci are pseudocolored in green, nuclei in blue. Scale bars: 20 μm; >100 cells were analyzed for each group. Mean ± SD is plotted. Two-tailed unpaired *t* test. For R-loops, serial dilutions starting from 500 ng DNA. Blue methylene (BM) staining was used as loading control. Bars represent mean ± SD of S9.6 signal quantification normalized on relative BM. One-way ANOVA test.

To narrow down splice variants leading to the phenotype observed in *SF3B1*-MT cells, we evaluated the overlap between commonly mis-spliced RNAs and targets identified in published DNA:RNA interactome studies (25–27). From this overlap, we identified several candidate RNA-binding proteins (RBPs) of interest, including *SERBP1*, *STAU1*, *SKIV2L*, and *THOC1*. *SERBP1* (SERPINE mRNA–binding protein 1) and *STAU1* (Staufen double-stranded RNA–binding protein 1) were common to all data sets (Figure 5A). *SERBP1* depletion has been reported to affect proper chromosome segregation during mitosis (28). *STAU1* is involved in various aspects of RNA metabolism, such as splicing, transport, decay, and translation (29). We also considered *SKIV2L* (Ski2-like RNA helicase) as a promising candidate as it was linked to R-loop biogenesis in 2 interactome and regulatome data sets (26, 27). Moreover, the alternative splice variants of *SERBP1* and *SKIV2L* are also conserved in *Sf3b1*-MT mice (5, 30). Functional characterization of *SERBP1*, *STAU1*, and *SKIV2L* proteins in the regulation of R-loops is not well explored. Among the common hits between *SF3B1* mutation–associated splice variants and R-loop regulatory proteins (26), we selected *THOC1* (THO complex 1) as a positive control because of its well-characterized role in maintaining R-loop homeostasis (31, 32). Notably, splice variants of these genes all resulted from alternative usage of 3′ splice sites involving either inclusion of part of an intron (*THOC1*, *SKIV2L*) or a non-canonical exon (*SERBP1*, *STAU1*) (Supplemental Figure 8, C and D). These alternative splicing events alter important functional domains for RNA binding/processing (*STAU1*, *SKIV2L*, *SERBP1*) or localization (*THOC1*), which might also impact the stability of the protein (Supplemental Figure 8C). By using reverse transcriptase qPCR, we validated the alternative splicing of these gene transcripts in the isogenic cell lines and human CLL cells harboring *SF3B1* mutations (Supplemental Figure 8, E and F).

To evaluate the role of splice variants in R-loop regulation, we used both shRNAs and sgRNAs to specifically knock down (KD) the genes of interest (Supplemental Figure 9, A–C). KD of the 4 RBPs examined all promoted both increased DNA DSBs (Figure 5, B and C, and Supplemental Figure 9D) and excessive R-loops (Figure 5, D and E, and Supplemental Figure 9E). KD of these genes in WT cells also phenocopied *SF3B1*-MT cells with impaired rates of transcription as measured by nuclear incorporation of EU (Supplemental Figure 9, F and G). Altogether, these results strongly support the notion that *SF3B1* mutation–associated splice variants of *SERBP1*, *SKIV2L*, *THOC1*, and *STAU1* contribute to reduced transcription rates and R-loop accumulation. Notably, the RBPs taken into consideration in our functional analysis act at various RNA processing steps ranging from transcription (*THOC1*) to mRNA stability/processing (*SERBP1*) and degradation (*SKIV2L*, *STAU1*), highlighting R-loop accumulation as a well-coordinated regulatory process in *SF3B1*-MT cells. Hence, our results overall suggest that dysregulated RNA splicing contributes to *SF3B1* mutation–associated R-loops.

### SF3B1 mutation modulates R-loop metabolism through SERBP1 alternative splicing.

**Figure 6:**
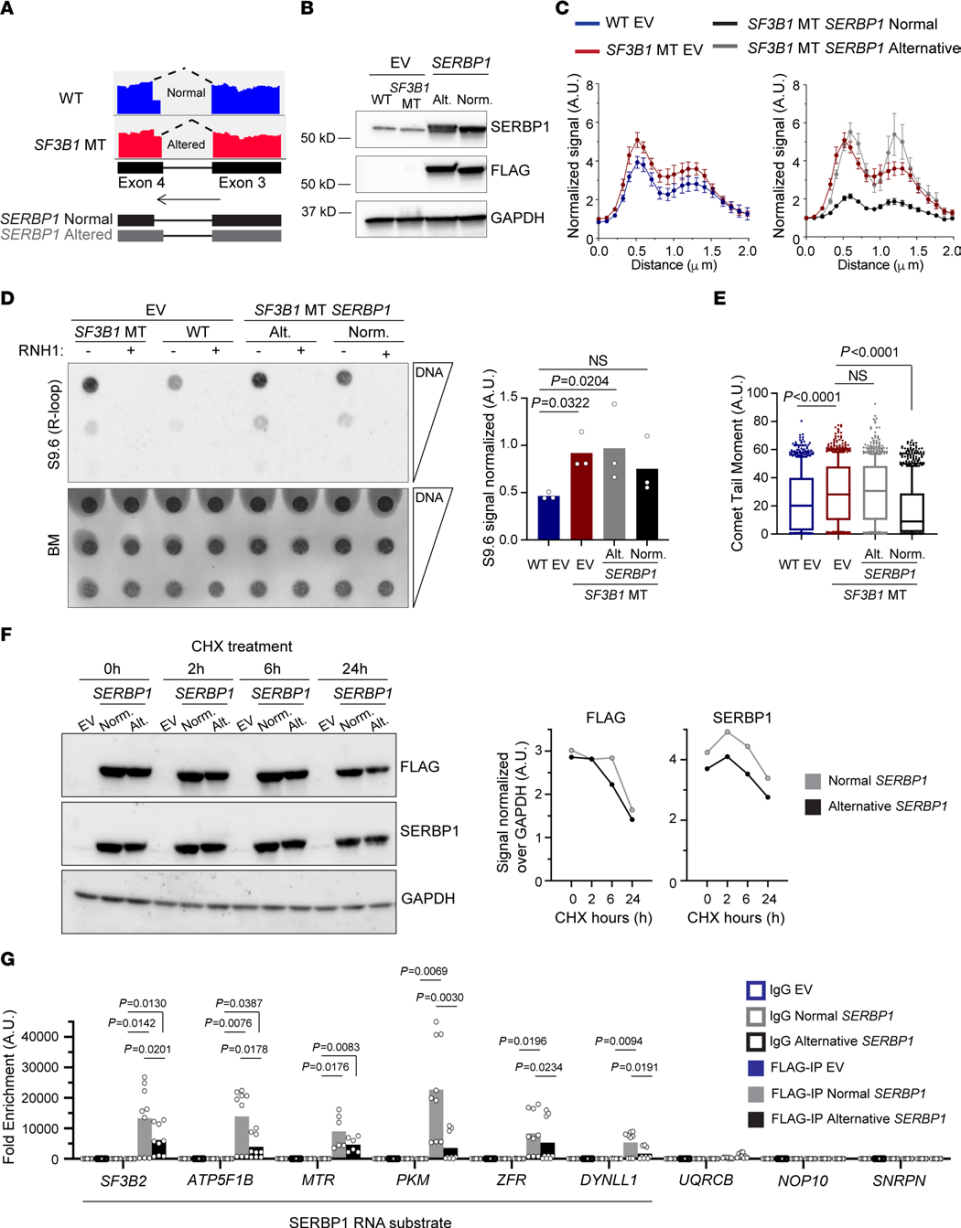
*SF3B1* mutation modulates R-loop metabolism through *SERBP1* alternative splicing. (**A**) IGV of RNA-Seq reads covering the cryptic 3′ splice site of the *SERBP1* gene in *SF3B1*-WT and -MT Nalm-6 cells. (**B**) Immunoblots of HEK293T *SF3B1*-WT and -MT cells overexpressing either FLAG empty vector (EV) or *SERBP1* FLAG-tagged isoforms. (**C**) Quantification of centromeric p-RPA immunofluorescence signal normalized to background near centromeres from cells described in **B**. *SF3B1*-MT EV cell line results are reported in 2 different graphs for better visualization. Wilcoxon’s paired test. HEK293T WT EV vs. *SF3B1*-MT EV, *P* < 0.0001; *SF3B1*-MT EV vs. *SF3B1*-MT *SERBP1* normal isoform, *P* < 0.0001; *SF3B1*-MT EV vs. *SF3B1*-MT *SERBP1* alternative isoform, *P* = NS. The number of chromosomes quantified ranges from 39 to 50. (**D**) Representative R-loops (left) and relative quantification (right) from dotblot assay in cells from **B**. Bars represent mean; dots represent biological replicates. One-way ANOVA comparison test. (**E**) Alkaline comet assay in cells as in **B**. Box plots show the median and 25th and 75th percentiles, with whiskers extending to minimum and maximum values. One-way ANOVA Dunnett’s multiple test. Comets quantified range from 783 to 995. (**F**) Left: Representative immunoblot of HEK293T cells as in **B**, treated for the indicated times with cycloheximide (CHX). Right: FLAG and SERBP1 immunoblot quantification normalized over GAPDH. (**G**) eCLIP-qPCR performed with HEK293T cells transfected as in **B**. *SF3B2*, *ATP5F1B*, *MTR*, *PKM*, *ZFR*, and *DYNLL1* were selected based on SERBP1 predicted mRNA target and R-loop–forming genes associated with *SF3B1* mutation. *SNRPN*, *NOP10*, and *UQRCB* were selected as negative controls.

*SERBP1* alternative splice variants are conserved in cell lines, murine B cells, and human CLL cells harboring *SF3B1* mutation (15, 30) (Figure 6A and Supplemental Figure 8, E and F). Through qPCR analysis, we estimated the relative expression for each isoform in our *SF3B1-*WT and -MT cell line panel and found that the most abundant isoforms are isoforms 3 and 4 (data not shown); the normal isoform of *SERBP1* is isoform 4, while the major alternatively spliced isoform is isoform 3 (Figure 6A and Supplemental Figure 8C). As *SERBP1* is a common hit among the R-loop interactomes studies, and its splice variants are associated with *SF3B1* mutation, we evaluated the impact of *SERBP1* on cen-R-loop abundance. By measuring aberrant mitosis, we found a higher frequency of cells with defective chromosome segregation upon *SERBP1* KD (Supplemental Figure 9H). Line-scan analysis of p-RPA and ACA costaining on chromosomal spreads revealed accumulation of cen-R-loops in *SERBP1*-KD cells similar to that in *THOC1*-KD cells (Supplemental Figure 9, I and J). Thus, our results suggest that R-loop–mediated mitotic stress is a common mechanism among cells with RNA processing defects. Importantly, overexpression of the canonical, but not the alternative, isoform of *SERBP1* (Figure 6, A and B) in *SF3B1*-MT cells alleviated excessive R-loops, centromeric p-RPA accumulation, and DNA DSBs (Figure 6, C–E, and Supplemental Figure 10A), strongly implicating direct involvement of *SERBP1* in the regulation of R-loop homeostasis.

At the protein level, we detected a reduction of SERBP1 in *SF3B1*-MT cells (Supplemental Figure 9, A and B). We hypothesized that SERBP1 protein expressed from the non-canonical variant might be less stable, and as a consequence the total amount of the protein decreased. To test this hypothesis, we examined the protein stability by expressing a FLAG-tagged cDNA of *SERBP1* normal and alternative isoforms and treating cells with cycloheximide (CHX). SERBP1 alternative isoform showed a faster reduction under CHX treatment compared with the normal one (Figure 6F). We also observed that the *SERBP1* non-canonical isoform altered the HABP-like domain of the protein (HABP4: intracellular hyaluronan-binding protein 4) (Supplemental Figure 8C), which likely would impact its RNA binding activity. To examine this, we determined transcriptome-wide RNA binding of SERBP1 using published cross-linking and immunoprecipitation sequencing (CLIP-seq) data (33). We found that SERBP1 preferentially bound to 154 mRNAs (Supplemental Figure 10B), mostly near the transcription starting site and within the coding sequence (Supplemental Figure 10C). Moreover, to determine the RNA binding sites of SERBP1 and their relationship with the R-loops associated with *SF3B1* mutation, we overlapped peaks from SERBP1 CLIP-seq data and DRIP-seq data (Supplemental Figure 10, D and E). We found more overlapping peaks (60) in *SF3B1*-MT than in WT cells (34) (Supplemental Figure 10D). Next, we examined the RNA binding efficiency of the 2 isoforms of SERBP1 by performing CLIP-qPCR (35) (Figure 6G and Supplemental Figure 10F). We selected 6 genes representing RNA targets of SERBP1 and loci of R-loop formation (*SF3B2*, *ATP5F1B*, *MTR*, *PKM*, *ZFR*, *DYNLL1*) (Figure 6G and Supplemental Figure 10, D and E); as control, we included 3 genes that were not targets of SERBP1 and not prone to form R-loops (*UQRCB*, *NOP10*, *SNRPN*) (Figure 6G). Canonical SERBP1 isoform bound all the expected targets, while the alternative displayed significant impaired binding to the same mRNAs (Figure 6G). Taken together, our results indicate that *SF3B1* mutation generates an alternative splice variant of *SERBP1*, encoding a less stable protein with impaired RNA binding, which in turn affects R-loop homeostasis.

### Primary CLL cells with SF3B1 mutations have R-loop dysregulation.

**Figure 7:**
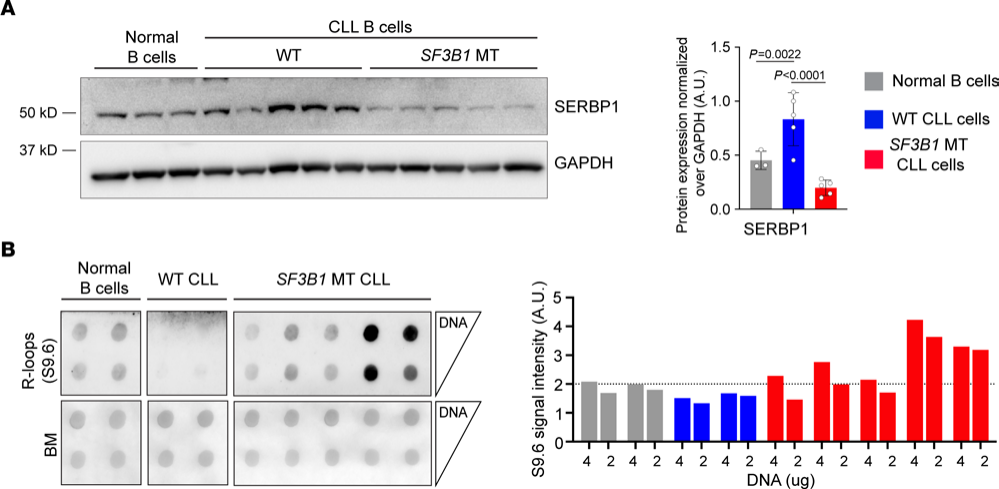
*SF3B1*-mutant CLL cells have R-loop dysregulation. (**A**) Left: Representative immunoblot of SERBP1 in normal (*n* = 3), CLL *SF3B1*-WT (*n* = 5), and *SF3B1*-MT (*n* = 5) B cells. Right: Relative immunoblot quantification normalized over GAPDH. Bar graphs represent data mean ± SD; dots represent biological replicates. Student’s 2-tailed *t* test followed by Bonferroni’s post hoc test. (**B**) Left: Dotblot assay for R-loop quantification in normal (*n* = 2), CLL *SF3B1*-WT (*n* = 2), and CLL *SF3B1*-MT (*n* = 5) B cells. Serial dilution of DNA starting from 4 μg. BM staining was used as loading control. Right: Relative dotblot S9.6 signal quantification normalized over BM signal. Each bar represents 1 biological replicate.

We further examined the level of SERBP1 and R-loops in primary human CLL samples with and without *SF3B1* mutations along with normal B cells from healthy donors. Altered splicing of SERBP1 (Supplemental Figure 8E) was reflected in a significantly lower level of SERBP1 protein expression in CLL *SF3B1*-MT cells compared with CLL WT and normal B cells (Figure 7A). R-loop measurements using dotblot assay indicated that *SF3B1*-MT CLL cells had an overall higher level of R-loops compared with normal B cells or CLL cells without *SF3B1* mutations (Figure 7B). These results reinforce the idea that *SF3B1* mutation dysregulates R-loop homeostasis through SERBP1 downregulation mediated by aberrant splicing in CLL.

### Sf3b1 mutation and Atm deletion cooperate to promote CLL development through cen-R-loop accumulation.

ATM has been shown to have a non-canonical role in spliceosome regulation (13, 34). Specifically, spliceosomes are displaced from stalling RNAPII, which causes R-loop accumulation and activates ATM to impede spliceosome organization upon formation of transcription-blocking DNA lesions (13). Consistent with this, we have reported that conditional heterozygous deletion of *Atm* in B cells increases intron retention of genes involved in DDR and cell cycle regulation (5). Here, we showed that genes encoding an R-loop–interacting protein are more likely to undergo aberrant RNA splicing in cells with *Atm* deletion and *Sf3b1* mutation (double mutant [DM]) than in cells with single mutation or with none (Supplemental Figure 10G). Furthermore, in cells with *ATM* deletion and *SF3B1* mutation (DM), expression of the aberrant splice variants (*THOC1*, *SERBP1*) was higher, resulting in reduced protein abundance (Supplemental Figure 10H). These results suggest that *ATM* deletion further impedes R-loop formation via aberrant RNA splicing of proteins involved in R-loop homeostasis.

**Figure 8:**
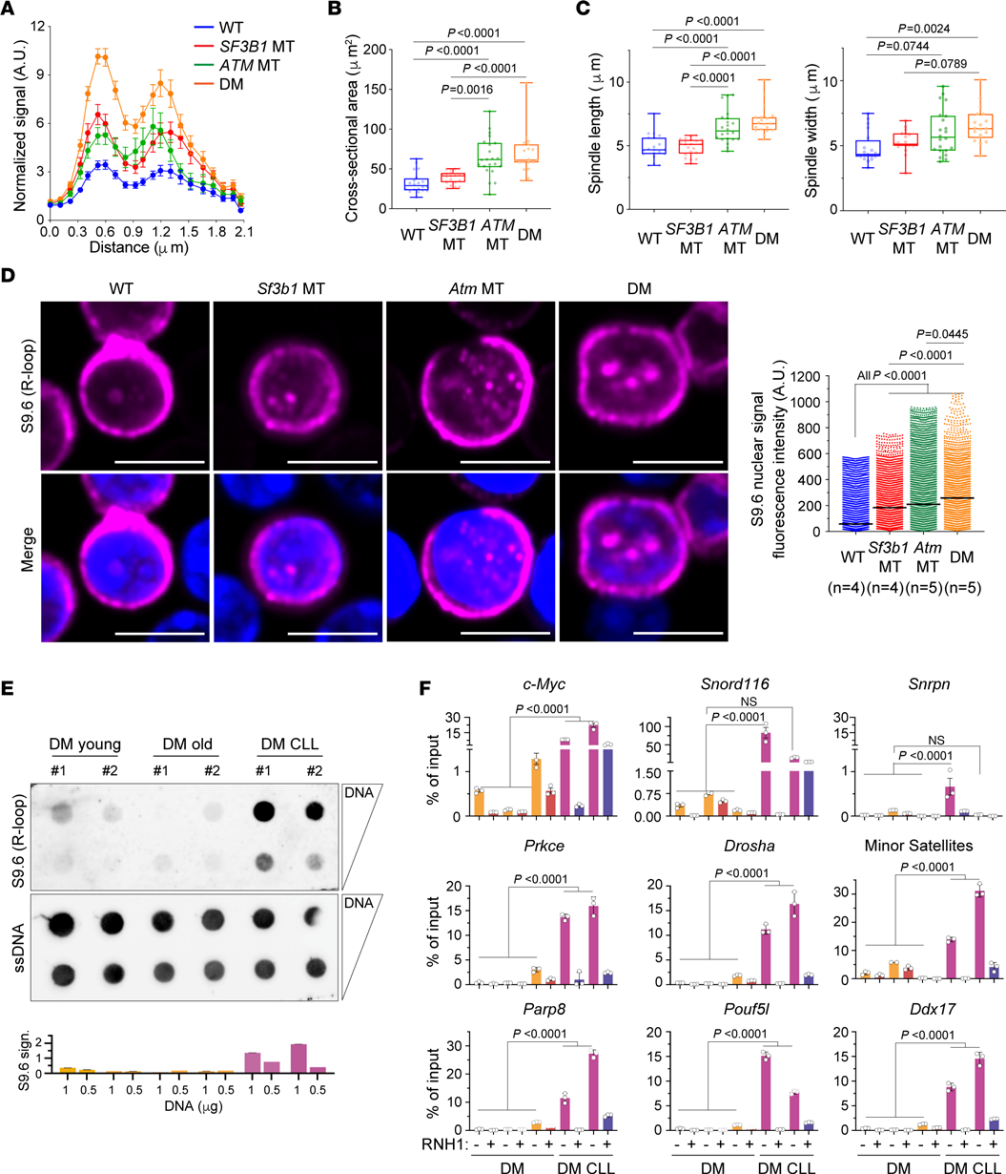
R-loop accumulation is a feature of murine CLL with *Sf3b1* mutation and *Atm* deletion. (**A**) Quantification of centromeric p-RPA signal in Nalm-6 Cas9 *SF3B1*-WT and -MT cells with and without *ATM* knockdown. Two-tailed paired *t* test, Nalm-6 WT vs. *SF3B1* MT, or vs. *ATM* MT, or vs. DM, *P* < 0.0001; *ATM* MT vs. DM, *P* < 0.0001; *SF3B1* MT vs. DM, *P* < 0.0001. The number of chromosomes quantified ranges from 56 to 113. (**B** and **C**) Quantification of 2-dimensional cross-sectional area of the entire body of chromosomes (**B**) and spindle length and width (**C**) in metaphases of cells described in **A**. Box plots show the median and 25th and 75th percentiles, with whiskers extending to minimum and maximum values. Dots represent biological replicates. Two-tailed unpaired *t* test followed by Bonferroni’s post hoc test. (**D**) Left: Representative images of R-loops detected by IF with S9.6 antibody (red) in WT, *Sf3b1*-MT, *Atm*-deleted (MT), and *Sf3b1*-MT and *Atm*-deleted (DM) murine splenic B cells. Scale bars: 5 μm. Right: Quantification of S9.6 nuclear fluorescence intensity. Number of mice used for each genotype is indicated. The number of cells quantified ranges from 2135 to 3690. Center lines show the medians. Two-tailed unpaired *t* test followed by Bonferroni’s post hoc test. (**E**) Top: Dotblot assay using splenic B cells derived from DM mice without and with CLL. Bottom: Relative S9.6 signal quantification normalized over ssDNA signal. Each bar represents 1 biological replicate. (**F**) DRIP-qPCR analysis of R-loop enrichment over negative (*Snrpn*) and positive (*c-Myc*, *Snord116*) loci for R-loop accumulation, over representative genes (*Prkce*, *Drosha*, *Ddx17*, *Parp8*, *Pouf5l*, and *Akt*) and centromeric regions (minor satellites), in normal and CLL B cells derived from DM mice. RNH1 treatment is included as control. Data are presented as mean ± SEM (*n* = 3, technical replicates). One-way ANOVA Tukey’s test. Untreated vs. RNH1-treated is significant for all samples tested (*P* < 0.0001).

To determine the role of *ATM* deletion in cen-R-loop formation, we analyzed the cen-R-loop level in Nalm-6 *SF3B1*-WT and -MT cells, with and without *ATM* deletion. *ATM* deletion triggered an increased R-loop formation at centromeres as compared with that in WT cells, which was similar to the level detected in cells with only *SF3B1* mutation (Figure 8A). Cells with both *SF3B1* and *ATM* lesions showed a significantly higher level of cen-R-loops compared with all other cell lines. Consequently, *ATM* deletion augmented chromosomal oscillation (Figure 8B) and spindle length and width (Figure 8C), which is in line with a previous observation that *ATM* depletion causes altered mitotic spindle structure (36, 37). More importantly, DM cells displayed more highly impaired mitotic fidelity compared with WT and single-lesion cells (Figure 8, A–C), confirming a synergy between the 2 lesions in promoting cen-R-loop accumulation.

Given that altered R-loop biogenesis is one of the underlying mechanisms for cancer development (38, 39), we hypothesized that *Sf3b1* mutation and *Atm* deletion synergistically lead to CLL development through R-loop accumulation. To address this, we first quantified R-loops in primary resting splenic B cells with *Sf3b1* mutation and/or *Atm* deletion via detection of nuclear S9.6 IF signals (Figure 8D). *Atm* deletion markedly increased nuclear R-loop levels compared with WT (*P* < 0.0001). DM murine B cells showed consistent R-loop accumulation compared with cells with either lesion (Figure 8D), suggesting a synergy between these 2 genetic lesions in disrupting proper R-loop formation/clearance from the genome. In our murine model, CLL typically develops as the mice age, phenocopying human CLL. By dotblot assay, we thus next investigated R-loop abundance in splenic B cells derived from young DM mice (3 months old) and old DM mice (24 months old) either without or with CLL (DM CLL). DM CLL cells exhibited a remarkable accumulation of R-loops in comparison with B cells, WT or with either lesion, while no difference was detected between young and old DM cells (Figure 8E). We confirmed this observation by assessing R-loop enrichment at different genes in murine DM B cells with (*n* = 2) and without CLL (*n* = 3) (Figure 8F). Compared with normal DM B cells, DM CLL cells exhibited substantially increased R-loops across all genes tested (*Akt3*, *Drosha*, *Ddx17*, *Prkce*, *Parp8*, *Pouf5l*), as well as in positive R-loop–forming regions (*Snord116*, ref. 40; and *c-Myc*, ref. 41), while no signal in all samples was detected over the negative control (*Snrpn*, ref. 40) and under RNH1 treatment (Figure 8F).

Murine DM CLL cells displayed substantial CIN compared with DM normal B cells (5). To clarify the contribution of dysregulated cen-R-loops to leukemogenesis, we next assessed R-loop levels formed over centromeric regions (minor satellite sequences) in DM B cells with and without CLL by DRIP-qPCR. Strikingly, DM CLL B cells exhibited significantly higher cen-R-loop accumulation compared with normal DM B cells (*P* < 0.0001; Figure 8F). These data demonstrate that R-loops are indeed regulated differentially in normal and leukemic cells, suggesting a contribution of enhanced cen-R-loop levels, driven by *Sf3b1* and *Atm* lesions, to CIN and CLL leukemogenesis in vivo.

**Figure 9:**
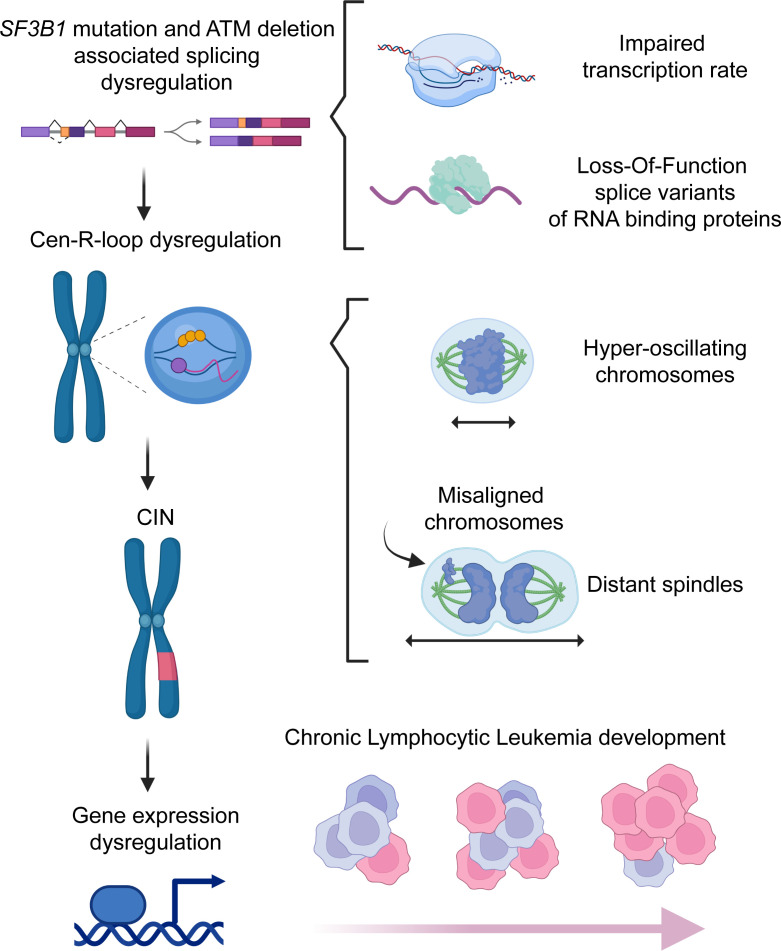
Working model. *SF3B1* mutation and *ATM* deletion together promote cen-R-loop accumulation, impairing mitotic spindle dynamics and chromosome alignment, resulting in CIN and CLL development. *SF3B1* mutation promotes global and centromeric R-loop formation through RNAPII transcription rate impairment and RBP loss-of-function alternative splice variants. Cen-R-loops alter mitotic spindle dynamics and chromosome segregation, resulting in CIN. *ATM* deletion in *SF3B1-*MT cells exacerbates cen-R-loops and CIN, promoting CLL development. Created with BioRender (biorender.com).

These observations led us to propose a working model for R-loop accumulation during CLL development (Figure 9). Specifically, *Sf3b1* mutation and *Atm* deletion trigger global and centromeric R-loop accumulation by generating splice variants in genes involved in R-loop biogenesis. Failure of ATM recruitment (due to deletion) to resolve R-loops exacerbates DNA damage and ultimately results in CIN. These genetic copy number changes further lead to dysregulated gene expression and, eventually, to CLL development.

## Discussion

Murine models with conditional heterozygous expression of splicing factor (SF) mutations (*SF3B1*, *U2AF1*, *SRSF2*, *ZRSR2*) have confirmed the causative effects of RNA splicing dysregulation in the pathogenesis of myeloid malignancies and CLL (5, 30, 42–45). However, the underlying molecular mechanism of how these SF mutations contribute to leukemogenesis remains elusive. In this study, we report that *SF3B1* mutation triggers genomic instability through excessive global and centromeric R-loop accumulation and *ATM* deletion further exacerbates the dysregulation of this process, ultimately resulting in CIN and possibly contributing to the initiation of leukemias.

Our results have several implications. First, our study highlights *SF3B1* mutation–associated cen-R-loop accumulation as a potential cancer driver. Although prior studies have reported that mutations in *SF3B1*, *SRSF2*, and *U2AF1* cause global R-loop augmentation (11, 12), to our knowledge, no studies thus far have assessed cen-R-loop levels or their roles in the pathogenesis of SF mutant leukemia. Recent studies revealed that cen-R-loop accumulation is emerging as a key regulator for suppressing CIN (10, 46). Our studies clearly demonstrate that maintaining a physiologic level of cen-R-loops is crucial in preventing CIN. *SF3B1*-MT cells accumulate high levels of cen-R-loops that result in increased frequency of aberrant mitosis. Treatment with RNH1 downregulates cen-R-loops in *SF3B1* MT to a level similar to that in WT cells (Figure 1F), leading to a reduced level of oscillating chromosomes and aberrant mitosis. These results reinforce the multifaceted nature of R-loops, highlighting the importance of maintaining their homeostasis.

Second, our study underscores that *SF3B1* mutation exerts its impact on R-loop homeostasis via RNA splicing–dependent and –independent mechanisms. *SRSF2* mutation was previously reported to induce RNAPII pausing in an RNA splicing–independent mechanism to promote R-loop accumulation (11); however, other SF mutations have unknown mechanisms for R-loop formation. R-loops are known to arise not only after collision between DNA replication forks and transcription machinery but also secondary to sub-stoichiometric levels of RNA-binding proteins (RBPs) coating the newly synthesized RNA (47). Deletion of *SFPQ* (splicing factor proline and glutamine rich), another splicing factor, causes R-loop accumulation by extending the interaction of the RNA helicase DHX9 with RNAPII (48). Depletion of the THO/TREX RBP complex affects mRNA transcription and transport, triggering R-loop–dependent DNA damage (31). Moreover, DHX9 and THOC1 (part of the THO/TREX RBP complex) have been linked with cen-R-loop regulation (48, 49). In line with these findings, our current results suggest that *SF3B1* mutation–associated R-loop accumulation uses both RNA splicing–independent and –dependent mechanisms. Our EU incorporation assays support the notion that *SF3B1* mutation influences transcription rates (Supplemental Figure 7) owing to splicing-independent (i.e., RNAPII pausing) (23) and splicing-dependent mechanisms (as expression of splice variants also resulted in transcriptional rate changes). The RBP (*SERBP1*, *THOC1*, *SKIV2L*, *STAU1*) loss-of-function splice variants involved in R-loop homeostasis phenocopy *SF3B1* mutation–associated genome-wide R-loop accumulation, with some (*SERBP1*, *THOC1*) also showing cen-R-loop augmentation, highlighting a lack of RBPs coating the nascent RNAs as a splicing-dependent mechanism. Our validation of *ATM* deletion on these splice variants supports that *ATM* deletion synergistically works together with *SF3B1* mutation to contribute to CIN.

Third, our study supports the notion that altered R-loop stability and biogenesis play critical roles in leukemogenesis. Because of the dynamic nature of R-loop formation/resolution, in vivo modulation of this biological process is challenging. Harnessing normal and CLL murine B cells with different genetic lesions, we showed that accumulated R-loop formation is linked to genetic lesions as well as CLL, suggesting that high levels of R-loops contribute to the onset of CLL. As we also demonstrated increased cen-R-loop formation in K562 *SF3B1-*MT cells, we speculate that *SF3B1* mutation may also induce CIN via cen-R-loop accumulation that contributes to the pathogenesis of myelodysplastic syndrome (MDS). In support of this, studies with primary MDS samples unraveled that *SF3B1* mutations are associated with CIN (50). For instance, about 50% of MDS cases have cytogenetic abnormalities, and most of these abnormalities are gains or losses of chromosomal materials (50). *SF3B1* mutations often co-occur with del(5q) (2). Moreover, a recently published paper delineates the landscape of clonal hematopoiesis–related single-nucleotide variants/indels and copy number abnormalities (CNAs) in the BioBank Japan cohort, and it concludes that *SF3B1* mutations are associated with CNAs (51). Given that SF mutations are prevalent in MDS, we anticipate that future studies of cen-R-loops related to SF mutation in MDS will likely further validate our notion.

Finally, *SF3B1*-MT cells have been reported to be sensitive to ATR (12) and PARP inhibitors as a result of R-loop–induced replication stress (52), providing synergy with other treatments such as splicing inhibitors and ionizing radiation (12, 52). Similarly, *SF3B1-*MT cells have been shown to be sensitive to nonsense-mediated RNA decay inhibitors (53). Our study indicates that *SF3B1*-MT cells are compromised in their ability to regulate cen-R-loops and exhibit a tolerance for high levels of R-loops as well as for increased spindle pressure from hyper-oscillating chromosomes. This raises the question of whether this unique state of dysregulation can be exploited to target *SF3B1*-MT leukemia. Two recent publications highlight that aneuploid cancer cells are vulnerable to mitotic checkpoint inhibition (54, 55). In particular, aneuploid cells exhibited aberrant spindle geometry and dynamics, and kept dividing when the spindle assembly checkpoint (SAC) was inhibited, resulting in the accumulation of mitotic defects and in unstable and less-fit karyotypes (54). However, we did not observe any benefit of targeting the SAC in treating *SF3B1*-MT leukemia (data not shown). Further studies on regulators of global and cen-R-loops will likely provide novel insights for therapeutic targeting of leukemic cells bearing *SF3B1* mutation.

Altogether, our data unveil how defective RNA processing triggers R-loop–mediated mitotic stress, leading to aberrant chromosomal burden. Our results suggest that mitotic stress derived from cen-R-loop augmentation is a molecular mechanism underlying leukemogenesis.

## Methods

### Primary samples.

Peripheral blood mononuclear cells from healthy donors and CLL patients were isolated by Ficoll-Hypaque density gradient centrifugation. CD19^+^ B cells were isolated by immunomagnetic selection (Miltenyi Biotec). Murine B cell enrichment from splenocytes using a negative MACS B cell Isolation Kit (Miltenyi Biotec) was described previously (5).

### Cell culture, generation of cell lines, and reagents.

HEK293T/LentiX-293T, Nalm-6, K562 cell lines were cultured in DMEM (Sigma-Aldrich), RPMI, and IMDM medium (Invitrogen), respectively. All media were supplemented with 10% FBS and 1% penicillin/streptomycin. Media for Nalm-6 and K562 SpCas9 cell lines were supplemented with blasticidin (Invitrogen). All cell lines were validated as mycoplasma-free. Nalm-6 and K562 isogenic cell lines with *SF3B1*-K700K (WT) and *SF3B1*-K700E (MT) were provided by H3 Biomedicine (14). HEK293T *SF3B1-*WT and -MT isogenic cell lines were generated as described in Supplemental Figure 1.

RNaseH1 WT-V5 tag and WKKD-V5 tag from Addgene (111906 and 111905) were subcloned into pLVX-EF1α-IRES-ZsGreen (Clontech) with EcoRI and NotI enzymes. Stable Nalm-6 cells overexpressing RNaseH1 WT or WKKD were generated through spin-infection with lentivirus (see Supplemental Methods) followed by sorting with GFP and maintained with more than 90% GFP positivity. Stable Nalm-6 and K562 cells overexpressing SpCas9 (52962, Addgene) were generated by spin-infection with SpCas9 lentivirus and selected with blasticidin. *ATM*, *SERBP1*, and *THOC1* were stably knocked out in Nalm-6 Cas9 cells, and *SERBP1* in K562 Cas9 cells, by spin-infection with sgRNAs targeting to these genes, which were cloned in pLKO5.1 mCherry (Addgene plasmid 57822). sgRNA target sequences are listed in Supplemental Table 1. mCherry-positive cells were sorted and maintained with more than 90% mCherry positivity.

Short hairpin RNAs (shRNAs) targeting *ROSA26* (shCTRL) and the 3′-untranslated region (3′-UTR) of *THOC1*, *STAU1*, *SERBP1*, and *SKIV2L* were cloned in pLVX-U6-EF1A-mCherry backbone and purchased from VectorBuilder. shRNA target sequences are listed in Supplemental Table 1. *SERBP1* splice isoforms were PCR-amplified from cDNA and subcloned into the expression vector pLVX-TetON-puro. pCDNA3.1 GFP-RNaseH1 was provided by Elodie Hatchi (Dana-Farber Cancer Institute). RNaseH1-eGFP-dCas9 construct (139835, Addgene) was generated as previously described (19). RNaseH1 WKKD–eGFP–dCas9 construct was generated by introduction of point mutations to dRNaseH1-eGFP-dCas9 (139836, Addgene) construct using the Q5 site-directed mutagenesis kit (New England Biolabs) according to the manufacturer’s protocol with minor modification (15 minutes of incubation with KLD enzyme mix). For RNaseH1-eGFP-dCas9 overexpression experiments, sgRNA control and sgRNAs targeting the *Actin* locus (19) and targeting α-satellite repetitive sequences (20) were cloned in pLKO5.1 mCherry (57822, Addgene) with sgRNA scaffold optimized for dCas9 (56).

All the antibodies, lentivirus production and spin transduction, immunoblotting, and qPCR are described in detail in Supplemental Methods.

### Immunofluorescence imaging, chromosome oscillations, and spindle architecture analysis.

Immunofluorescence (IF) was performed as previously described with minor modifications (18). Briefly, cells were fixed in 4% paraformaldehyde (BioLegend) at room temperature (RT). After 20 minutes in fixative buffer, cells were rinsed in PBS, permeabilized in PBS 1X - 0.5% Trition X-100, washed twice, blocked with 1% BSA/PBS for 1 hour at RT, and incubated at 4°C overnight with the primary antibodies in blocking buffer. After 3 washes with PBS buffer, cells were incubated with the secondary antibodies in 1% BSA for 1 hour at RT in the dark. After 3 more washes with PBS, stained cells were mounted onto glass slides with Fluoroshield with DAPI (Sigma-Aldrich) for nuclear staining. Incubation with anti–α-tubulin antibody was performed for 2 hours at RT in PBS/1% BSA. For R-loop detection, cells were fixed and permeabilized with ice-cold 80% methanol at –20°C for 20 minutes, and cells were blocked in 5% BSA/PBS buffer. IF staining of Nalm-6, K562, Hg3, and murine splenic B cells was performed in suspension and followed by cytospin at 165 *g* for 5 minutes onto glass slides (Shandon Double Cytoslides, Thermo Fisher Scientific). Imaging was performed with an Olympus DP80 epifluorescent microscope or Zeiss LSM 880 with Airscan confocal microscope. Pictures were analyzed with QuPath (version 0.1.2). Chromosome oscillation and spindle geometry were analyzed as previously described (54, 55). Spindles and chromosomes were stained with FITC-conjugated monoclonal anti–α-tubulin antibody and DAPI, respectively. Images were collected by taking *Z*-stacks with a step size of 0.3 μm covering the entire volume of the mitotic cell. Metaphase spindle distance and width were measured after defining of the position of the 2 poles. The total distribution of chromosomes was measured by analysis of their relative area occupied. For each cell line, 20 metaphases were analyzed. Confocal images were analyzed with QuPath (version 0.1.2). Representative images presented in figures are maximum intensity projections of entire *Z*-stacks.

### Immunolabeling of unfixed metaphase spreads and line-scan analysis.

Labeling of metaphase spreads and relative line-scan analyses were performed as previously described with minor modifications (10). Chromosome spreads were obtained from cells that were pretreated with 100 ng/mL colcemide (Roche) for 30 minutes. Cells were subsequently washed with ice-cold PBS once and then resuspended in 75 mM KCl hypotonic solution for 10 minutes at 37°C before being spun onto glass slides (Shandon Double Cytoslides, Thermo Fisher Scientific) at 1,100 *g* for 10 minutes. Slides were incubated in KCM buffer (120 mM KCl, 20 mM NaCl, 10 mM Tris-HCl pH 8, 0.5 mM EDTA, 0.1% [vol/vol] Triton X-100) for 10 minutes. Primary antibodies were diluted in 1% BSA/KCM buffer and incubated with slides for 2 hours in a humid chamber at RT. Slides were then washed for 10 minutes in KCM buffer and incubated with secondary antibodies diluted in 1% BSA/KCM buffer 1 hour at RT in a humid chamber. Slides were subsequently washed with KCM buffer, fixed with 4% paraformaldehyde, and mounted with Fluoroshield with DAPI for DNA staining. All buffers used were supplemented with either 100 nM okadaic acid (10011490, Cayman Chemical) or phosphatase/protease inhibitor cocktail (Thermo Fisher Scientific) to prevent protein dephosphorylation and degradation. Analysis of chromosomes was conducted in Fuji ImageJ using the profile analysis function. A line was drawn through the centromeres using the ACA signal as reference. Fluorescence intensity was normalized to background immediately outside ACA signal. Fluorescence intensities were averaged across distance, and SEM was calculated per distance point. Chromosome pairs were randomly selected with a minimum total of 6 cells analyzed per condition.

### RNA/DNA hybrid dotblot and immunoprecipitation (DRIP).

Dotblot and DRIP experiments were carried out with DNA extracted from non-cross-linked cells using a NucleoSpin Tissue Kit (Macherey-Nagel). RNAseH1 (New England Biolabs) digestion was performed at 37°C for 1 hour. DNA was spotted on Amersham Hybond-N+ membrane (GE Healthcare) with a Bio-Dot apparatus (Bio-Rad Laboratories). After UV cross-linking, the membrane was stained with 0.02% methylene blue solution. The membrane was washed with TBS buffer, blocked with 5% nonfat milk in TBS-Tween 0.2%, and incubated with anti–DNA-RNA Hybrid Antibody (clone S9.6 MABE1095, Millipore) overnight at 4°C. For the loading control, the membrane was denatured for 10 minutes in 0.5 M NaOH, 1.5 M NaCl, neutralized for 2 minutes in 0.5 M Tris-HCl pH 7.2, 1.5 M NaCl, and reprobed with an anti-ssDNA antibody (Millipore) overnight at 4°C. Both anti–DNA-RNA Hybrid and anti-ssDNA were revealed with HRP-conjugated anti-mouse IgG secondary antibody and Clarity Western ECL Substrate (Bio-Rad Laboratories). Images were acquired with ChemiDoc MP (Bio-Rad Laboratories) and S9.6, Blue Methylene, and ssDNA signals quantified using ImageJ software (version 1.3.1).

DRIP was carried out as previously reported with modifications (21). DNA was digested by HindIII EcoRI, BsrGI, XbaI, and SspI (New England Biolabs) restriction enzyme cocktail. One percent of the DNA preparation was kept as input DNA. Half of the fragmented DNA was treated with RNase H (New England Biolabs) overnight at 37°C. DNA samples with or without RNase H treatment were then incubated with anti–DNA-RNA S9.6 monoclonal antibody (courtesy of Teresa V. Bowman, Albert Einstein College of Medicine) preblocked on Dynabeads Protein G beads (Thermo Fisher Scientific) in immunoprecipitation buffer (50 mM HEPES/KOH at pH 7.5; 0.14 M NaCl; 5 mM EDTA; 1% Triton X-100; 0.1% Na-deoxycholate, ddH_2_O) overnight at 4°C with rotation. Beads were washed twice with the following buffers sequentially: low-salt buffer (50 mM HEPES/KOH pH 7.5, 0.14 M NaCl, 5 mM EDTA pH 8, 1% Triton X-100, 0.1% Na-deoxycholate), high-salt buffer (50 mM HEPES/KOH pH 7.5, 0.5 M NaCl, 5 mM EDTA pH 8, 1% Triton X-100, 0.1% Na-deoxycholate), wash buffer (10 mM Tris-HCl pH 8, 0.25 M LiCl, 0.5% NP-40, 0.5% Na-deoxycholate, 1 mM EDTA pH 8), and TE buffer pH 8.0 (AM9849, Invitrogen, Thermo Fisher Scientific). Elution was performed in 100 μL of elution buffer (50 mM Tris-HCl pH 8, 10 mM EDTA, 1% SDS) for 20 minutes at 65°C. The DNA was purified by phenol-chloroform extraction and ethanol precipitation after every step of digestion and immunoprecipitation. The immunoprecipitated DNA recovered was analyzed either by qPCR or by next-generation sequencing. The next-generation sequencing library was constructed with a SMARTer ThruPLEX DNA-Seq kit for sample preparation (Takara Bio) and sequenced using a HiSeq2000 machine with 50-bp single-read protocol.

### DRIP-seq data analysis.

Raw sequencing reads were mapped to the human reference genome (GRCh38/hg38) using BWA-MEM with default parameter setting (57). Mitochondrial DNA was filtered using SAMTools (58), and duplicated reads were removed using Picard tools MarkDuplicates (http://broadinstitute.github.io/picard/). R-loop narrow peaks were called using MACS2 (59) with parameters “-B -f BAMPE -g -q 0.05.” Fragment size was estimated using MACS2 built-in model. Bona fide R-loop peaks were called over RNaseH1-treated samples and used for all the analyses. The R-loop peak list was then exported to ChIPseeker (60) to generate chromosome coverage plot and annotation for genomic location. For differential peak enrichment analysis, DiffBind (61) and DESeq2 (62) were used to identify regions that were differentially bound between mutant and WT groups. Peaks with fold change greater than 2 and FDR less than 0.05 were kept for further analysis and Circos plot visualization (63). Peak was visualized with Integrative Genomics Viewer (64).

### Comet assay.

DNA breaks were monitored using Single Cell Gel Electrophoresis Assay with CometAssay Reagent Kit (4250-050-K, Trevigen). Cells were combined with low-melting agarose at 37 °C at a 1:10 ratio of cells to agarose and spread onto CometSlide. After gelling in 4°C for 30 minutes in the dark, the slides were immersed in 4°C lysis solution overnight. Electrophoresis was performed at 4°C at 21 V for 30 minutes in either neutral or alkaline buffer. For neutral comet assays, slides were immersed in DNA precipitation solution for 30 minutes followed by 70% ethanol incubation for 30 minutes at RT. For alkaline comet assays, slides were incubated for 1 hour at 4°C in unwinding solution before electrophoresis and washed twice with ddH_2_O followed by 70% ethanol. Finally, slides were dried at 37°C for 10 minutes, and DNA was stained with SYBR Gold Nucleic Acid Gel Stain solution (Thermo Fisher Scientific). Slides were scanned with an Olympus DP80 epifluorescent microscope. Cells treated with DNA damage agents (doxorubicin, hydroxyurea) were included as control. At least 50 comets per sample were measured with the plug-in OpenComet for ImageJ software (version 1.3.1), and tail moment represents tail length and the fraction of DNA in the tail.

### Quantification of global RNA transcription rate.

Global RNA transcription rate was detected using 5-ethynyluridine (EU), Click-and-Go Plus 488 Imaging Kit, and Click-and-Go Plus 647 Imaging Kit (Click Chemistry Tools). Cells were seeded in a 6-well plate at a concentration 1 × 10^6^ cells/mL overnight and incubated with 1 mM EU for 30 minutes to label newly synthesized RNA. Nalm-6 cells and murine B cells were cytospun and mounted with coverslips, and slides were scanned using an Olympus DP80 epifluorescence microscope with the ×63 objective. EU incorporation in HEK293T cells with transfected shRNAs was evaluated by flow cytometry. Data were analyzed using FlowJo (version 10) software.

### RNA sequencing library construction and splicing analysis.

Total RNA was isolated using the Macherey-Nagel NucleoSpin RNA kit. The library was constructed using a KAPA stranded RNA-Seq kit (Roche) according to the manufacturer’s instructions. The paired-end 101-bp library was sequenced on Illumina Sequencing Platforms with 50 million reads per sample. RNA splicing analysis was performed as we previously described (65). For differential splicing analysis of each cell line, we adopted the differential splicing analysis statistical model from rMATS (66) with absolute IncLevelDifference value greater than 0.1 and FDR less than 0.05 as significance cutoff (Supplemental Table 2).

### Enhanced UV cross-linking and RNA immunoprecipitation coupled with qPCR.

Enhanced UV cross-linking and RNA immunoprecipitation (eCLIP) was performed based on a previously described protocol (34). Twenty million HEK293T cells with overexpression of normal or alternative *SERBP1* isoforms were resuspended in ice-cold PBS buffer (1.2 × 10^6^/mL) and cross-linked twice with 254 nM UV cross-linker (200 mJ/cm^2^). Cells were lysed in 800 μL lysis buffer (50 mM Tris-HCl pH 7.4, 100 mM NaCl, 1% NP-40, 0.1% SDS, 0.5% sodium deoxycholate) with 1.6 μL SUPERase•In RNase Inhibitor (AM2694, Invitrogen) and sonicated with 6 cycles at 10 seconds on/20 seconds off per cycle, 30% amplitude (Thermo Fisher Scientific). Cell supernatant was digested by 3.3 μL RNase I (AM2295, Invitrogen) and 2 μL DNase I (Invitrogen, AM2239) on a ThermoMixer (Eppendorf) at 37°C with shaking at 1,100 rpm for 3 minutes to remove DNA and free RNA. Cell supernatant was equally divided for control and anti-FLAG reactions. Two percent of each reaction was kept as pre-immunoprecipitation input. The lysed samples were mixed with beads-antibody complex for immunoprecipitation (4°C, 1 hour). Four micrograms anti-FLAG antibody (F1804, Sigma-Aldrich) or anti-mouse IgG (31878, Invitrogen) and 25 μL Protein G Dynabeads (10003D, Invitrogen) were premixed for each reaction at RT for 1 hour. After immunoprecipitation, the beads-protein-RNA complexes were washed 3 times with 400 μL high-salt wash buffer (50 mM Tris-HCl pH 7.4, 1 M NaCl, 1 mM EDTA pH 8.0, 1% NP-40, 0.1 SDS, and 0.5% sodium deoxycholate) at 4°C for 2 minutes. Each reaction was split into 2 identical parts at the third wash step, one part for protein-bound RNA analysis and the other for immunoprecipitation-immunoblotting verification. After removal of the supernatant, 1 mL of TRIzol Reagent (15596026, Invitrogen) was added into tubes for RNA elution. Bolt LDS Sample Buffer (1×; B0008, Invitrogen) was used for protein elution from the beads-protein-RNA complexes. The eluted RNAs and proteins including pre-immunoprecipitation input samples were subjected to qPCR and immunoblotting analysis, respectively.

### Statistics.

Statistical analysis was performed using GraphPad Prism 9.01. *P* values were calculated with Student’s *t* test, Wilcoxon’s test, or 1-way ANOVA test. When there are multiple comparisons, *P* values were corrected by Bonferroni’s post hoc test. One-tailed *t* test was applied where required to specifically assess a predefined direction of the biological effect under consideration. The type of statistical test used and the results including *P* value, means, median, and standard error are shown in the figures and legends.

### Study approval.

Specimens were collected from CLL patients in accordance with the principles of the Declaration of Helsinki and with the approval of the Institutional Review Boards of City of Hope (Duarte, California, USA). Written informed consent was obtained from all patients. All animals were housed at City of Hope. All animal procedures were completed in accordance with the *Guide for the Care and Use of Laboratory Animals* (National Academies Press, 2011) and were approved by the Institutional Animal Care and Use Committees at City of Hope.

### Data and materials availability.

RNA-Seq (HEK293 cells) and DRIP-seq (Nalm-6 cells) data were deposited in the NCBI’s Gene Expression Omnibus database (GEO GSE235208). The RNA splicing analysis pipeline is published (66). All other data needed to evaluate the conclusions in the paper are in the supplemental material. Request of reagents and protocols should be submitted to LW.

## Author contributions

MC and LW designed the study. MC designed, performed, and analyzed all experiments, except for S9.6 immunofluorescence on murine splenic B cells, analyzed by AL. HS performed Western blot from Nalm-6 ATM cell and CLL samples, dotblot from CLL samples, SERBP1 isoform stability, and eCLIP. BZ, KH, and PI maintained animal cohorts. MC and YW generated sgRNAs targeting *THOC1* and *SERBP1* constructs and lentivirus. MF generated *SERBP1* normal and alternative splice variant overexpression constructs. PI generated K562 *SF3B1*-WT and -MT cell lines overexpressing SpCas9. YW generated Nalm-6 *RNaseH1-*WT and -WKKD cell lines. LW and KH generated the total RNA libraries. MJ performed RNA-Seq alternative splicing analysis. MJ and LY performed DRIP-seq analysis. LY performed eCLIP-seq analysis. TVB, SN, EAO, SMPM, JS, CG, CJW, and RJL contributed to experimental materials and suggestions. LW and MC prepared the manuscript with help from all coauthors.

## Supplementary Material

Supplemental data

Supplemental table 1

Supplemental table 2

Supporting data values

## Version 1 07/18/2023

In-Press Preview

## Version 2 09/01/2023

Electronic publication
