# Supplementary material for: SF3B1 mutation and ATM deletion codrive leukemogenesis via centromeric R-loop dysregulation
Source: J Clin Invest. 2023 Sep 1;133(17):e163325. doi: 10.1172/JCI163325 (PMC10471171; doi:10.1172/JCI163325)
Supplement: Supplemental data [file jci-133-163325-s031.pdf]

Supplemental Methods:

Lentivirus production and spin infection

LentiX-293T cells (Clontech, Mountain View, CA) were plated on 6-well plates at 3×10^5 cells per well in DMEM medium with 10% FBS and allowed to adhere over-night. Transfections were performed with 5 µg of gene expression plasmid along with 1.5 µg psPAX2, and 0.4 ug pVSV-G packaging plasmids per manufacture instructions with polyethylenimine method (PEI Max 40K #24765, Polysciences, Warrington, PA) in serum-free Opti-MEM media (Life Technologies, Thermo Fisher Scientific Inc, Grand Island, NY). After 24 hours, cell media was replaced with fresh complete DMEM with 10 % FBS and the cells were incubated for an additional 24 to 48 hours for virus production. Supernatant was collected and filtered through a 0.45 µM Nalgene syringe filter SFCA (Whatman, Clifton, NJ), and virus was concentrated by ultracentrifugation in 38.5 mL tubes (#344058, Beckman Coulter, Brea, CA) at 22000 x g for 2 hours at 4°C. For spin-infection, 0.5 million cells were transduced with 50µl of concentrated virus and centrifuged at 2200 rpm for 90 minutes at 37°C, along with a final concentration of polybrene at µg/ml (#TR-1003-G, Millipore Sigma, Billerica, MA).

Antibodies

Anti-GAPDH G-9 (sc-365062), anti-THOC1 E-10 (sc-514123), anti-SAP62 A-3 (sc-390444), anti-SAP49 G-3 (sc-365571), anti-beta-Actin C4 (sc-47778), were purchased from Santa Cruz Biotechnology (Dallas, Texas). Anti-γH2AX (JBW301 05-636), anti-phospho-H3Ser10 (06-570), anti-DNA-RNA Hybrid Antibody (clone S9.6 MABE1095) were purchased from Millipore (Sigma, Billerica, MA). Anti-V5-tag D3H8Q (#13202S), anti-Cas9 (S. pyogenes) D8Y4K (#65832S), anti-ATM D2E2 (#2873S) were purchased from Cell Signaling (Danvers, MA). Anti-RPA(S33) (#A300-246A) was purchased from Bethyl laboratories (Montgomery, TX). Isothiocyanate (FITC)-conjugated monoclonal anti-α-tubulin antibody (#F2168-.2ML) was purchased from Sigma, (St. Louis, MO). Anti-DNA-RNA hybrid S9.6 (Kf-Ab01137-23.0) was purchased from Kerafast (Boston, MA). Human anti-centromere antisera (ACA) (15-234-0001) was purchased from Antibodies Inc. (Davis, CA). Alexa Fluor 488-AffiniPure Goat anti-Human IgG (H+L) (#109-545-088) was purchased from Jackson ImmunoResearch Laboratories (West Grove, PA). DYKDDDDK tag monoclonal antibody (#MA191878), Alexa Fluor 488 Donkey anti-Rabbit

(#A21206) and anti-Mouse (#A21202), Alexa Fluor 647 Donkey anti-Rabbit (#A10042) and anti-Mouse (#A10037), anti-SERBP1 (#PA5-54235) were all purchased from ThermoFisher Scientific (Waltham, MA). Monoclonal S9.6 antibody generated from HB-8730 hybridoma used for RNA: DNA hybrid immunoprecipitation was kindly provided by Teresa Bowman Laboratory from Albert Einstein College of Medicine (Bronx, NY).

Western blotting

Cells were harvested, washed once with PBS, and lysed for 30’ at 4°C with RIPA buffer (ThermoFisher Scientific, Waltham, MA) supplemented with a cocktail of phosphatase/protease inhibitors (Pierce™ Protease and phosphatase inhibitor minitablets EDTA-free, ThermoFisher Scientific, Waltham, MA) before sonication. Cell debris were removed by centrifugation at 10,000×g for 30’, and protein quantification was evaluated with BCA assay (Pierce™, ThermoFisher Scientific, Waltham, MA). For immunoblot analysis, proteins extracts were fractionated by SDS-PAGE (4-15% Criterion Precast Gel, Bio-Rad laboratories, Hercules, CA; 3-8% NuPAGE Tris-Acetate Gel, ThermoFisher Scientific, Waltham, MA) and transferred to Trans Blot Turbo nitrocellulose membranes (Bio-Rad laboratories, Hercules, CA). After blocking with 5% non-fat dried milk, membranes were stained with primary antibodies and appropriate secondary antibodies. Bands revealed by Western ECL Substrate (Bio-Rad laboratories, Hercules, CA). Images were acquired with ChemiDoc MP (Bio-Rad laboratories, Hercules, CA). Protein band signals were quantified using ImageJ software (version 1.3.1).

Quantitative PCR

Total RNA was extracted from cell lines and human CLL cells using Trizol (ThermoFisher Scientific, Waltham, MA) followed by DNAseI (New England BioLabs, Ipswich MA) digestion and ethanol purification. 500 ng of RNA was transcribed into cDNA using High Capacity cDNA Reverse Transcription Kit (ThermoFisher Scientific, Waltham, MA). QPCR was performed with either PowerUp™ SYBR™ Green Master Mix (Applied Biosystems, Carlsbad, CA, A25741) or iTaq™ Universal SYBR® Green Supermix (Bio-Rad laboratories, Hercules, CA, 1725124), and analyzed on QuantStudio™ 7 Flex Real-Time PCR System (Applied Biosystems, Carlsbad CA). Target gene expression was normalized to the mean Ct values of the housekeeping gene *GAPDH*.

Splice variant expression was normalized to the mean Ct values of the normal isoform. Primers sequences were included in Supplemental Table 1.

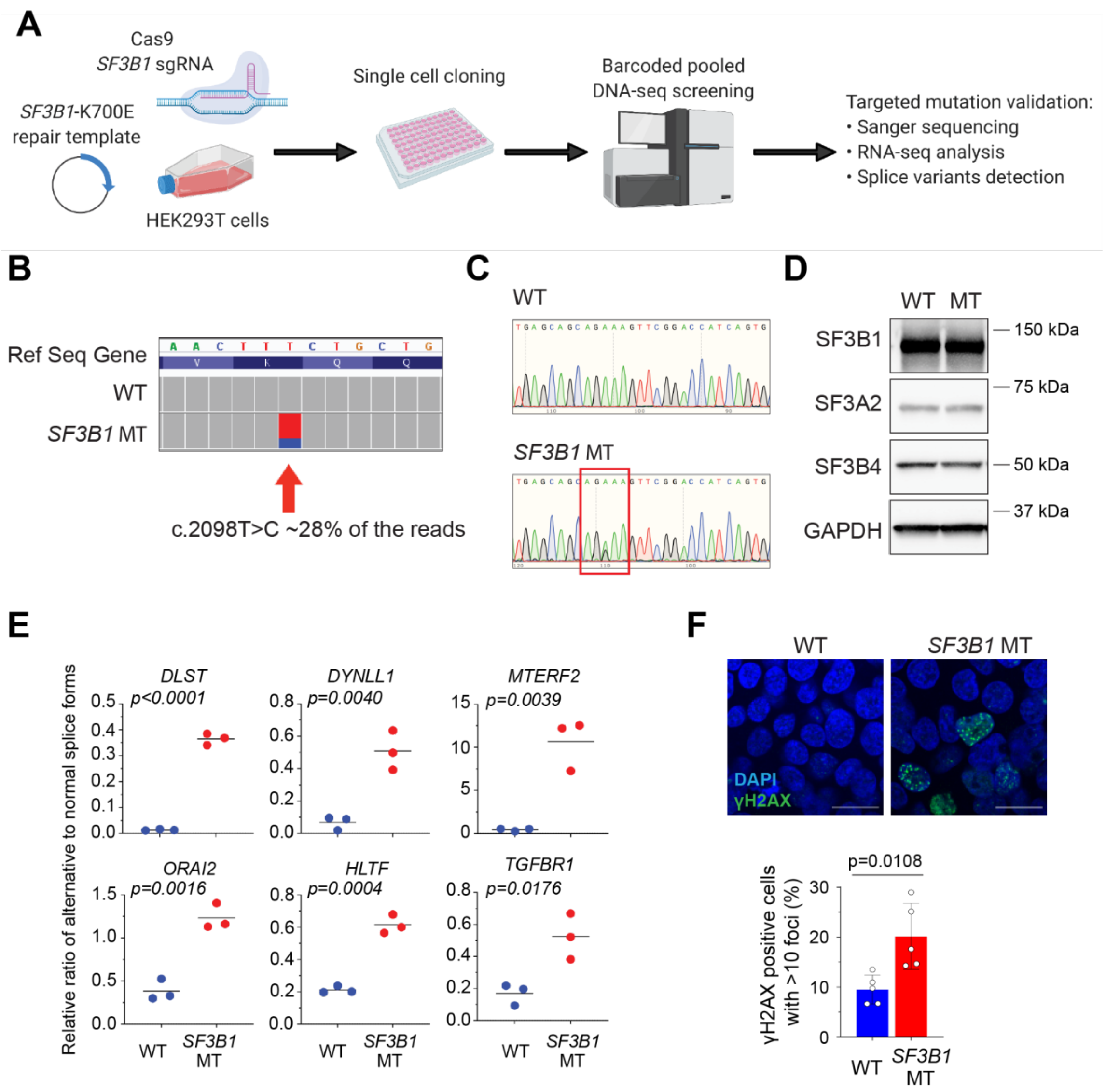

Supplemental Figure 1. Generation of HEK293T *SF3B1* mutant isogenic cell line. (A) Strategy to generate HEK293T isogenic cell line with *SF3B1*-K700E at the endogenous locus using the CRISPR/Cas9 system. Cartoon generated with BioRender.com. **(B)** DNA-seq reads covering *SF3B1*-K700E site. **(C)** Sanger sequencing of targeted mutation region in HEK293T *SF3B1* WT and MT clones. **(D)** Western blot analysis of spliceosome components SF3B1, SF3A2, and SF3B4 in HEK293T *SF3B1* WT and MT cells. GAPDH used as loading control. **(E)** qPCR analysis of 3'ASS splice variants associated with *SF3B1* mutation. **(F)** γH2AX immunofluorescence (top) and relative quantification (bottom) in HEK293T *SF3B1* WT and MT. γH2AX foci pseudocolored in green, nuclei in blue. Scale bar: 20µm. Each dot represents the mean of cells with more than 10 γH2AX foci in biological replicate. Graphs represent data mean ± SD. Two-tailed *t*-test.

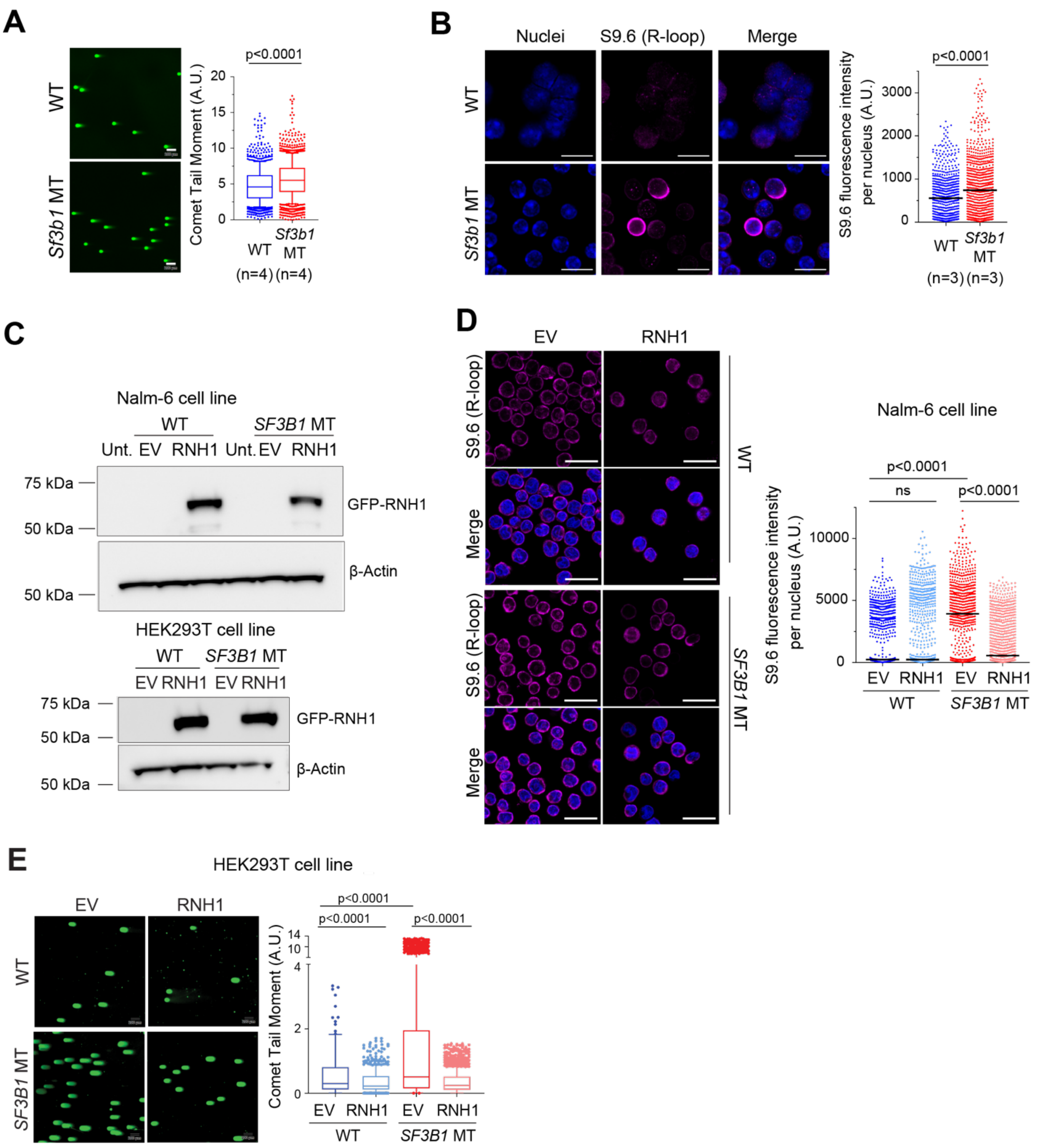

Supplemental Figure 2. RNaseH1 over-expression alleviates R-loop accumulation and DNA damage in *SF3B1* MT cells. (A) Left: representative images of neutral comet assay for DNA double stranded breaks of murine splenic B cells with (MT) or without *Sf3b1* mutation (WT) (left). Scale bar: 100μm. Right: quantification of relative comet tail moment from *Sf3b1* WT (n=902) and MT (n=1022) cells. **(B)** Representative images of R-loops immunofluorescence staining with S9.6 antibody in murine splenic *Sf3b1* WT (n=916), and MT (n=1137) B cells (left). Nuclei staining with DAPI (blue). Scale bar: 20μm. Quantification of S9.6 nuclear fluorescence intensity (right). Center lines show the medians. In panel **(A)** and **(B)**, the number of mice for each genotype

used is indicated. **(C)** Detection of GFP tag with immunoblot in *SF3B1* WT and MT Nalm-6 cells (top) and HEK293T cells (bottom) with over-expression of either empty vector (EV) or RNAseH1 (RNH1) GFP-tagged constructs. **(D)** Left: Representative images of R-loop presence detected by immunofluorescence staining with S9.6 antibody (pseudocolored in red) in Nalm-6 *SF3B1* WT and MT cells with either over-expression of EV or RNH1 GFP-tagged constructs. DAPI staining used to mark nuclei (blue). Scale bar: 20μm. Right: relative quantification of nuclear S9.6 immunofluorescence signal intensities (EV WT (n=731); RNaseH1 WT (n=889); EV *SF3B1* MT (n=760); RNaseH1 *SF3B1* MT (n=840)) in three experiments. Center lines show the medians. Two-tail *t*-test. **(E)** Left: representative images of DNA damage detected by neutral comet assay in HEK293 *SF3B1* WT and MT cells with over-expression of either GFP-EV or GFP-RNH1 construct. Scale bar: 100μm. Right: quantification of neutral comet tail moment (EV WT (n=126); RNH1 WT (n=198); EV *SF3B1* MT (n=96); RNH1 *SF3B1* MT (n=163)). Center lines show the medians, box limits indicate the 25th and 75th percentiles, and whiskers extend to minimum and maximum values. Two-tailed *t*-test.

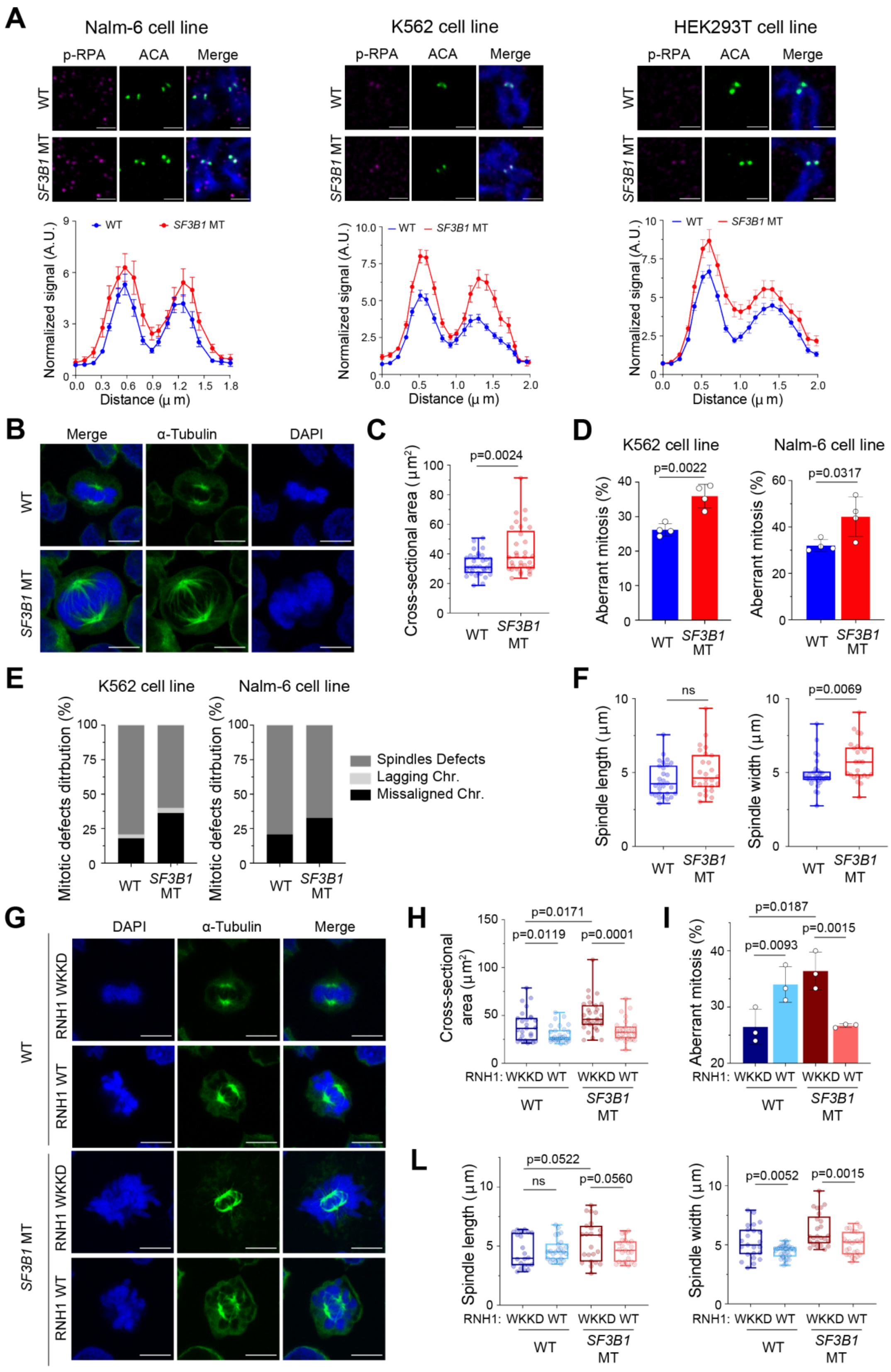

Supplemental Figure 3. Mitotic stress in *SF3B1* mutant cell lines. **(A)** Centromeric R-loops

detected by co-staining of phospho-RPA S33 (p-RPA) and centromere (ACA) using immunofluorescence (IF) staining on chromosome spreads in indicated cell lines. On top of all panels, representative images of p-RPA and ACA IF. Chromosomes marked with DAPI, pseudocolored in blue. Scale bar: 2µm. Asynchronously growing cells were arrested for 30 minutes with colcemide (100ng/ml) to facilitate chromosome spreading. On bottom of all panels, quantification of centromeric p-RPA immunofluorescence signal normalized to background near centromeres (Methods). Error bars represent data mean ± SEM. The number of chromosomes quantified: Nalm-6 *SF3B1* WT (n=67), *SF3B1* MT (n=66); K562 *SF3B1* WT (n=118), *SF3B1* MT (n=110); HEK293T *SF3B1* WT (n=140), *SF3B1* MT (n=141). Two-Tailed paired *t*-test for all three cell types: *SF3B1* WT *vs.* MT p-value <0.0001. **(B)** Representative confocal images of chromosome area and alignment (DAPI staining, pseudocolored in blue), and mitotic spindles (α-Tubulin staining, pseudocolored in green) of *SF3B1* WT and MT Nalm-6 cells in metaphase. Scale bar: 5µm. **(C)** Quantification of two-dimensional cross-sectional area of the entire body of chromosomes in more than 20 *SF3B1* WT and MT Nalm-6 cells in metaphase. **(D)** Aberrant mitosis frequency (lagging chromosomes/chromosome bridges, misaligned chromosomes, or spindle defects) in *SF3B1* WT and MT K562 and Nalm-6 cells expressed as % of total mitosis encountered. (**E**) Aberrant mitosis frequency distribution in *SF3B1* WT and MT K562 and Nalm-6 cells expressed as % of total aberrant mitotic process encountered. Bar graphs report data mean. More than 100 mitotic cells counted for each group. **(F)** Spindle length and width were measured from confocal images in more than 20 *SF3B1* WT and MT Nalm-6 cells in metaphase. The definitions of length and width are shown in Figure 5. (**G**) Representative confocal images of metaphase chromosome oscillation and spindle geometry as described in **(B)** of *SF3B1* WT and MT Nalm-6 cells with overexpression of either RNaseH1 (RNH1) WKKD or WT. Scale bar: 5µm. (**H**) Analysis of two-dimensional cross-sectional area of the entire body of chromosomes in Nalm-6 *SF3B1* WT and MT over-expressing either WKKD (WKKD) or WT RNH1 (WT). (**I**) Distribution of mitotic defects in *SF3B1* WT and MT Nalm-6 cells with overexpression of either RNH1 WT or WKKD form. (**L**) Spindles length (left) and width (right) analysis in cells from panel (**G**)**.** In panel (**D**), and (**I**) bar graphs represent data mean ± SD. Each dot represents a biological replicate. Two-tailed unpaired *t*-test. In panel (**C**), (**F**), (**H**), and (**L**) center lines show the medians, box limits indicate the 25th and 75th percentiles, and whiskers extend to minimum and maximum values. Two-tail *t*-test was used.

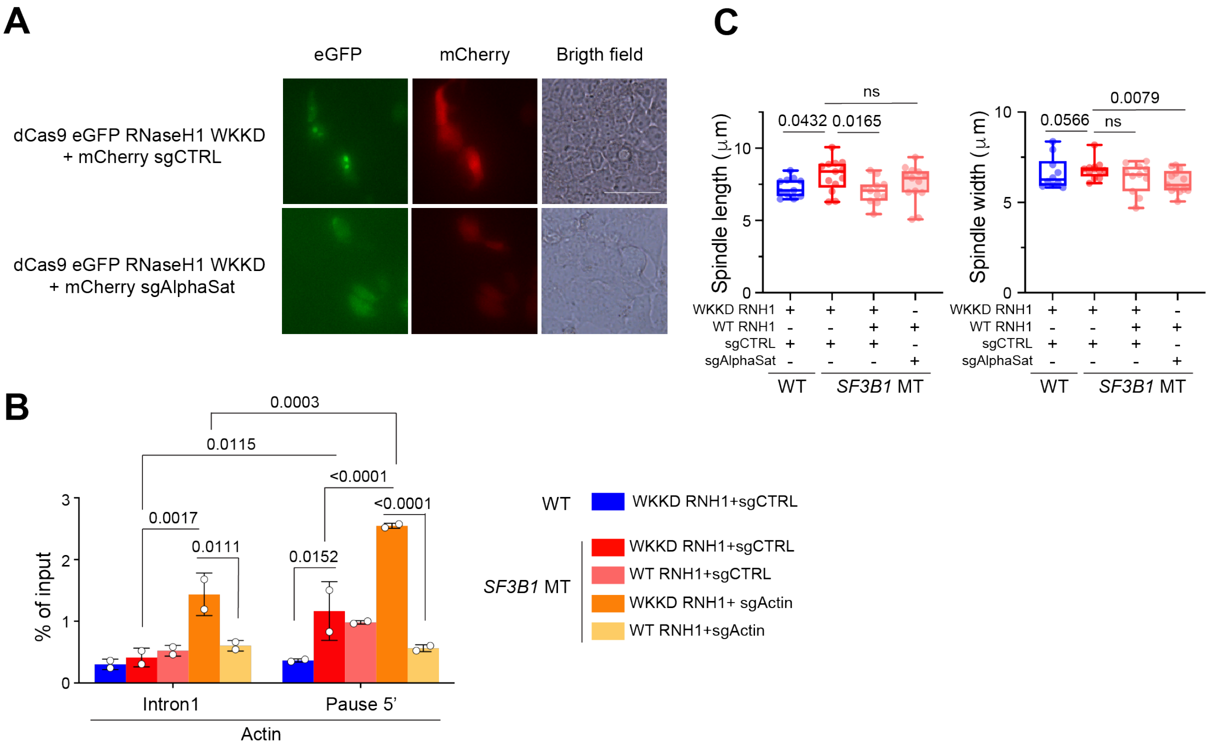

Supplemental Figure 4. dCas9 eGFP RNaseH1 modulation of R-loop. (**A**) Representative microscope images of HEK293T cells co-transfected with dCas9 eGFP RNaseH1 WKKD along with either sgRNA targeting to control region (sgCTRL) or centromeric repeats AlphaSatellite (sgAlphaSat). Scale bar: 50μm. (**B**) DRIP-qPCR in HEK293T *SF3B1* WT and MT over-expressing either dCas9-GFP-RNaseH1 WKKD (WKKD RNH1) or dCas9 GFP RNaseH1 WT (WT RNH1) in combination with either sgCTRL or a specific sgRNA targeting *Actin* locus (sgActin). Bar graphs represent data mean ± SEM. Two-tailed unpaired *t*-test. (**C**) Spindle length (left) and width (right) analysis in HEK293T *SF3B1* WT and MT over-expressing either dCas9-GFP-RNaseH1 WKKD (WKKD RNH1) or dCas9 GFP RNaseH1 WT (WT RNH1) in combination with either sgCTRL or sgAlphaSat. Center lines show the medians, box limits indicate the 25th and 75th percentiles, and whiskers extend to minimum and maximum values. Two-tail unpaired *t*-test was used.

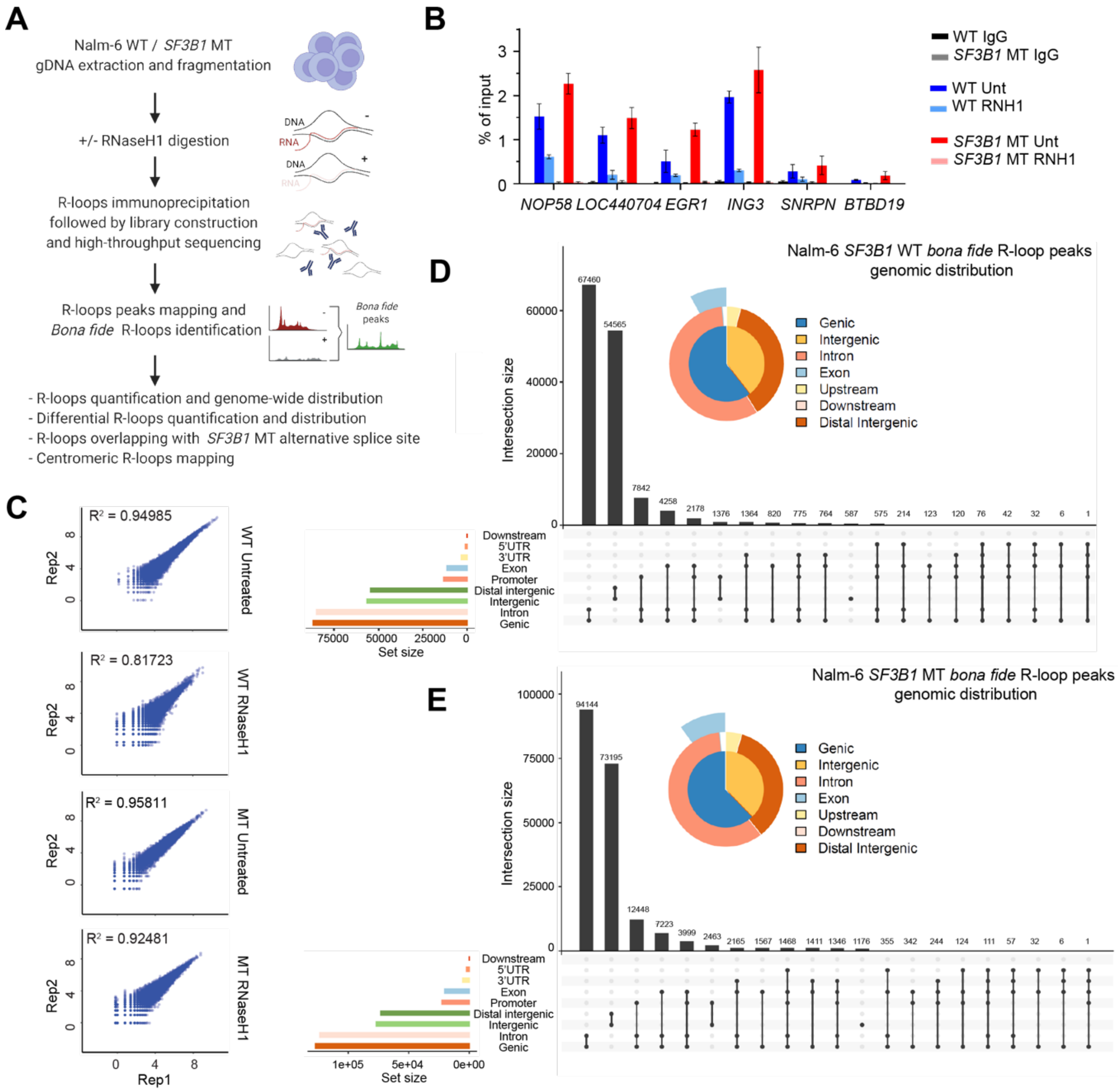

Supplemental Figure 5. DRIP-sequencing identifies *bona fide SF3B1* mutation-associated R-loops. (**A**) Schema of the DRIP-seq experiment. **(B)** Representative q-PCR to validate proper DNA:RNA hybrids immunoprecipitation (DRIP) in Nalm-6 cells with and without *SF3B1* mutation. *NOP58*, *LOC440704*, *EGR1*, and *ING3* genes were tested for R-loop accumulation. *SNRPN* and *BTDB19* genes were used for negative R-loop forming regions. RNaseH1 (RNH1) treated samples used as background control. Graphs represent qPCR result of biological duplicates; data are plotted as mean±SEM. **(C)** Pair-wise comparison of biological replicates for DRIP-seq library. Pearson correlation coefficient was computed from each comparison to evaluate the reproducibility. (**D**) and **(E)** Upset plots and PieChart plots show *bona fide* R-loop genomic distribution in Nalm-6 *SF3B1* WT (**D**) and MT (**E**) cells.

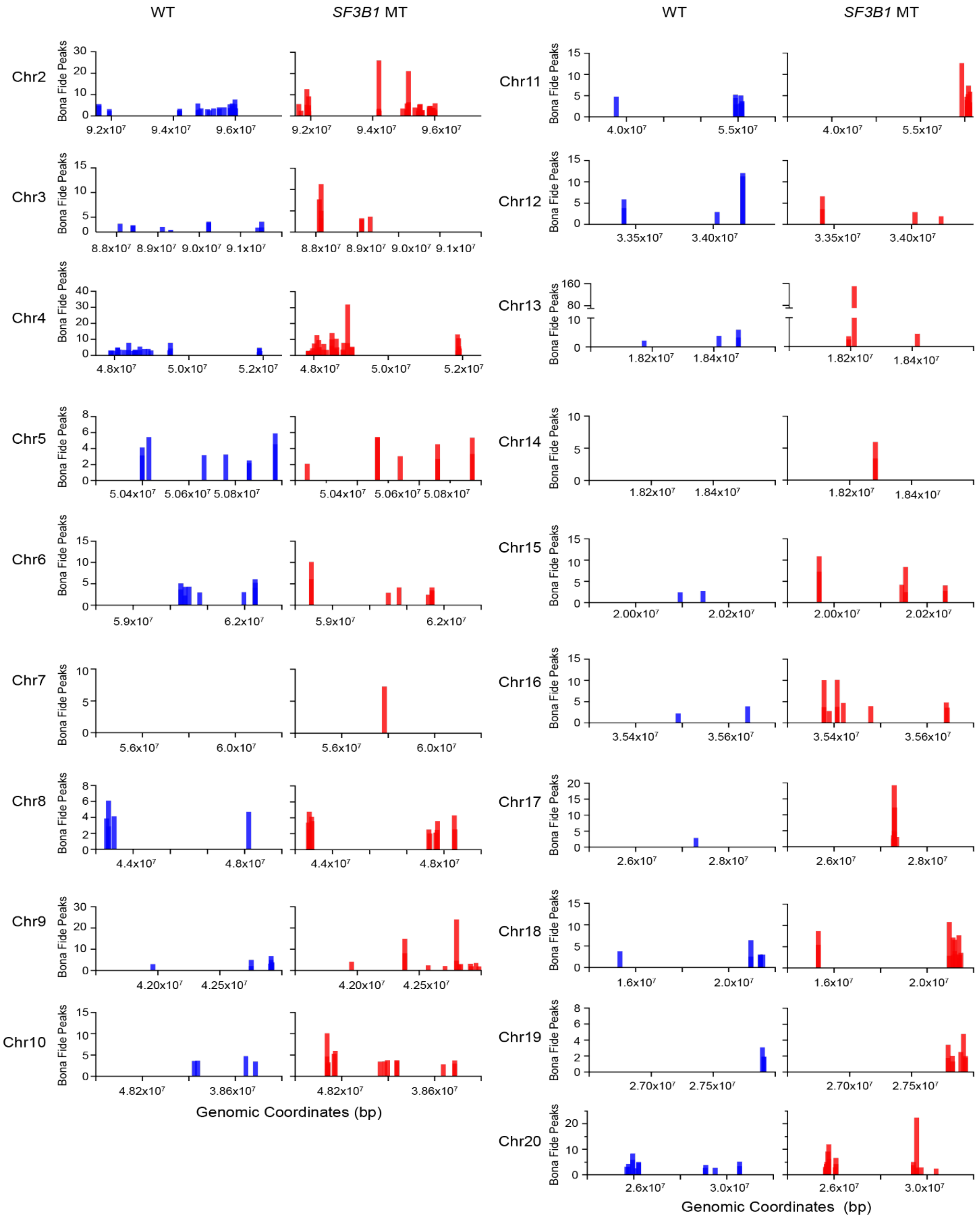

Supplemental Figure 6. R-loops peaks distribution on centromeric regions. Distribution of *bona fide* R-loops peaks identified with DRIP-sequencing and mapped at different chromosome centromeric region.

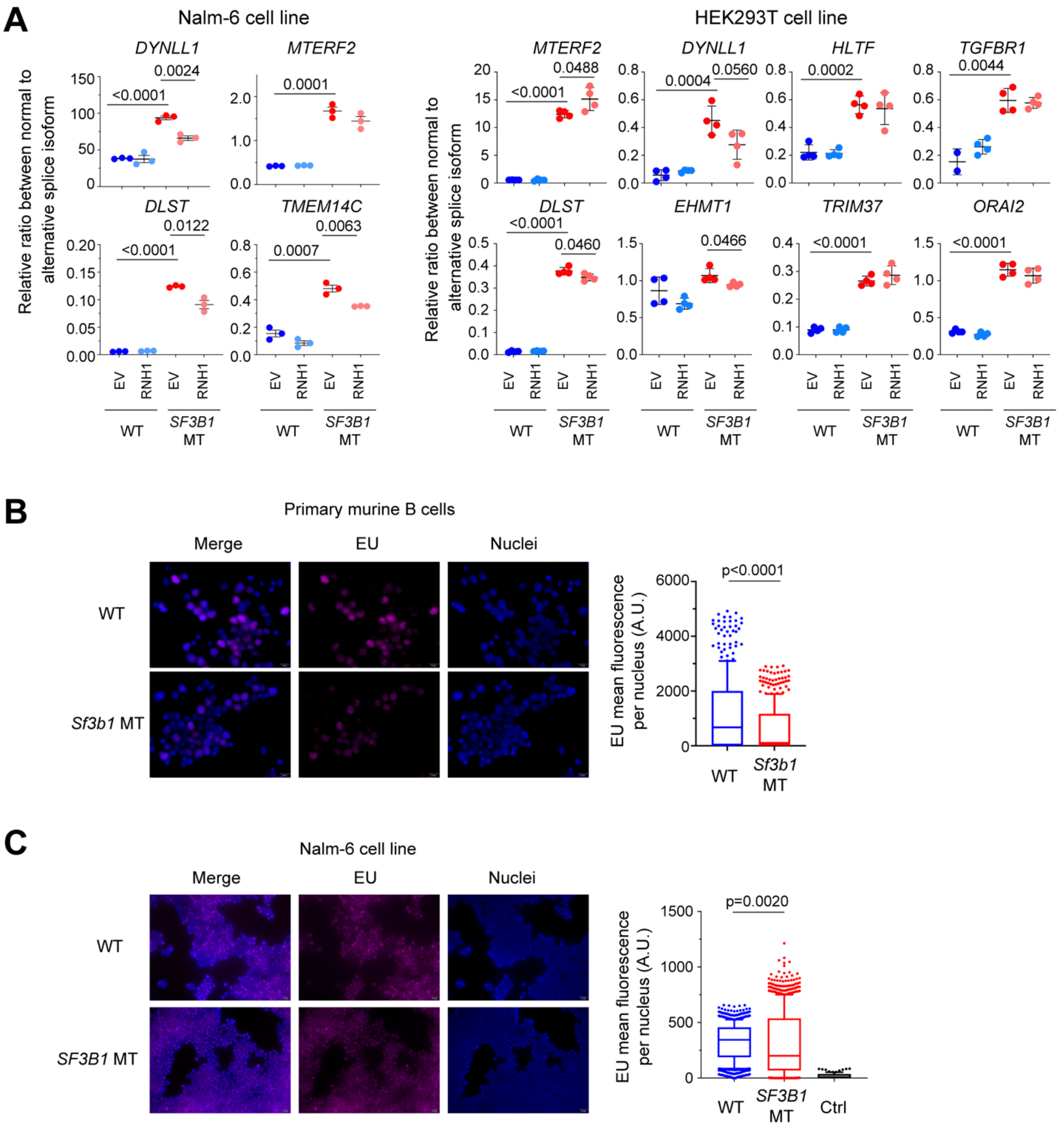

Supplemental Figure 7. *SF3B1* mutant cells have reduced RNA transcription rate. (A) Validation of impact of RNase H1 over-expression on selected *SF3B1* mutation associated splice variants in Nalm-6 (left) and HEK293T (right) WT and *SF3B1* MT cells, over-expressing either empty vector (EV) or RNase H1 (RNH1) construct by q-PCR. Graphs represent data mean±SEM, each dot represents a replicate. Two-tailed *t*-test. **(B)** and **(C)** Left: representative images of 5-ethynyl uridine (EU) incorporation in murine splenic B cells with or without *Sf3b1*-K700E mutation (**B**) and in Nalm-6 *SF3B1* WT and MT (**C**). Cells were incubated for 30 minutes with EU, subsequently

detected with Click-iT Nascent RNA capture, which is a click chemistry-based florescence detection method (Methods); DAPI marks nuclei (blue). Scale bar 20μm. Right: quantification of relative EU mean fluorescence per nucleus: in primary murine B cells WT (n=423), and *Sf3b1*-K700E (n=479) (**B**); in Nalm-6 *SF3B1* WT (n=931), and MT (n=939) (**C**). Whiskers plot's center lines show the medians, box limits indicate the 25th and 75th percentiles, and whiskers extend to minimum and maximum values. Two-tailed *t*-test. No EU (Ctrl) sample showed as control.

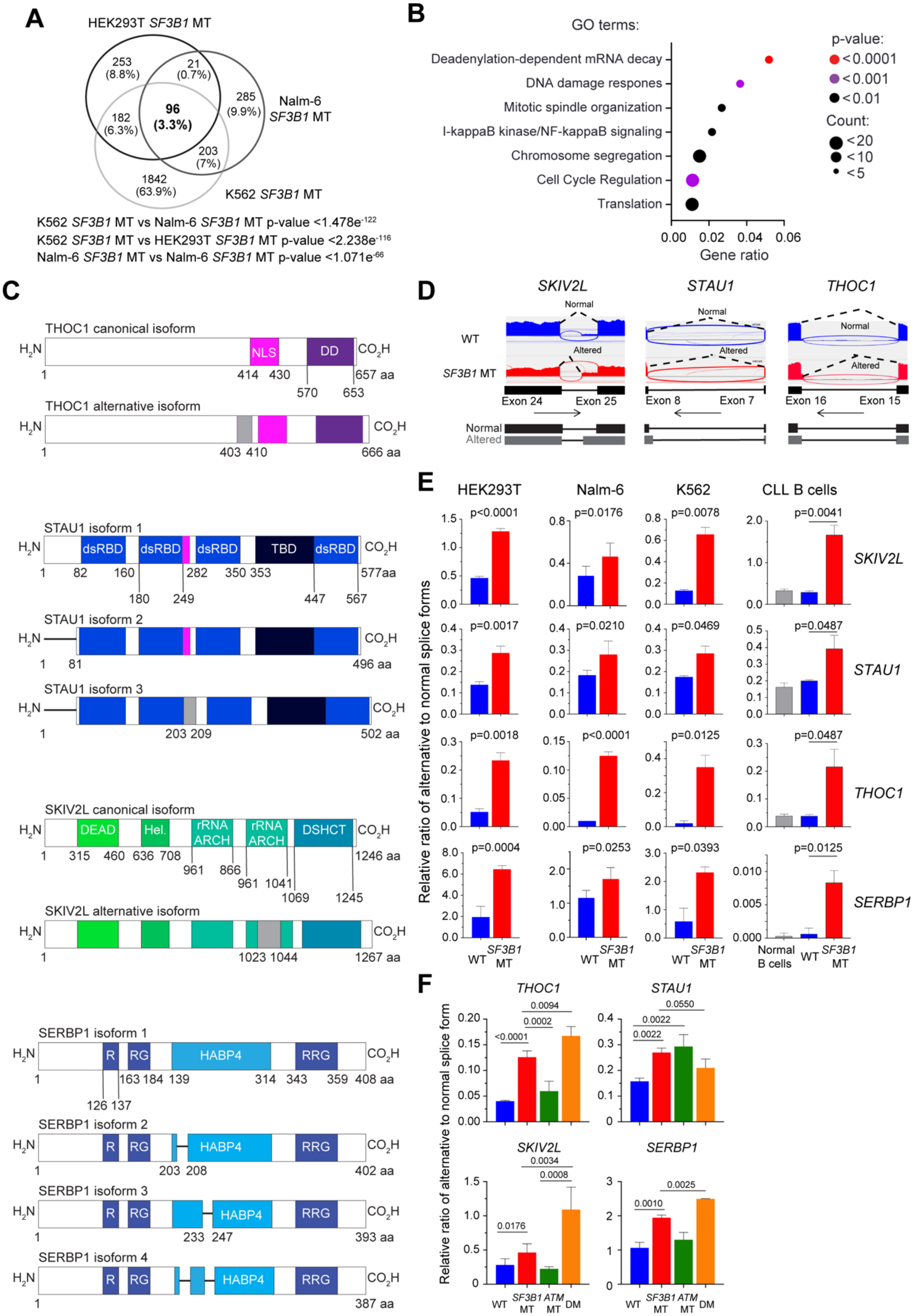

A
HEK293T SF3B1 MT
Nalm-6 SF3B1 MT
K562 SF3B1 MT
253 (8.8%)
21 (0.7%)
285 (9.9%)
182 (6.3%)
96 (3.3%)
203 (7%)
1842 (63.9%)
K562 SF3B1 MT vs Nalm-6 SF3B1 MT p-value <1.478e-122
K562 SF3B1 MT vs HEK293T SF3B1 MT p-value <2.238e-116
Nalm-6 SF3B1 MT vs Nalm-6 SF3B1 MT p-value <1.071e-66
B
GO terms:
Deadenylation-dependent mRNA decay
DNA damage respones
Mitotic spindle organization
I-kappaB kinase/NF-kappaB signaling
Chromosome segregation
Cell Cycle Regulation
Translation
Gene ratio
p-value:
<0.0001
<0.001
<0.01
Count:
<20
<10
<5
C
THOC1 canonical isoform
THOC1 alternative isoform
STAU1 isoform 1
STAU1 isoform 2
STAU1 isoform 3
SKIV2L canonical isoform
SKIV2L alternative isoform
SERBP1 isoform 1
SERBP1 isoform 2
SERBP1 isoform 3
SERBP1 isoform 4
D
SKIV2L
STAU1
THOC1
WT
SF3B1 MT
Normal
Altered
Exon 24
Exon 25
Exon 8
Exon 7
Exon 16
Exon 15
E
HEK293T
Nalm-6
K562
CLL B cells
Relative ratio of alternative to normal splice forms
SKIV2L
STAU1
THOC1
SERBP1
WT
SF3B1 MT
Normal B cells
F
THOC1
STAU1
SKIV2L
SERBP1
Relative ratio of alternative to normal splice form
WT
SF3B1 MT
ATM MT
DM

Supplemental Figure 8. ***SF3B1*** **mutation alters RNA splicing and impacts newly synthesized mRNA rate. (A)** Venn diagram of splice variants associated with *SF3B1* mutation in HEK293T, K562, and Nalm-6 isogenic cell lines. **(B)** Gene ontology analysis of shared splice variants identified from (**A**) using Metascape custom analysis (1) with the following criteria: minimum overlap: 2; p-value cut off: 0.05; minimum enrichment: 1.5. (**C**) Schematic cartoon representing simplified protein structure of RBPs *THOC1*, *STAU1*, *SERBP1*, *SKIV2L,* with different isoforms depicted along with known functional domains (www.uniprot.org; www.genecards.org). THOC1 presents two important domains, the nuclear localization signal (NLS, in pink) and a death domain (DD, in purple). The alternative isoform presents the insertion of 9 amminoacids R>SVCFVHDR in position 403 just before the NLS and the DD domain. The 9 amino acid insertion introduces a series of bulky amino acids that cause a change in the peptide strand direction, altering the NLS and DD domain conformation. This may lead to altered function, localization, or stability of THOC1 protein. STAU1 is involved in various aspects of RNA metabolism such as RNA localization, splicing, stability, translation, and decay(2). As result, STAU1 impact a wide variety of cell functions ranging from cell proliferation to migration, apoptosis, autophagy, and stress response. *STAU1* has two major isoforms(2). STAU1 presents four different double strand RNA binding domain (dsRBD, in blue), one NLS (in pink), and a tubulin binding domain (TBD, in black). Of the four dsRBD domains, the second and the third are required for direct binding of STAU1 to mRNAs, while the second and the fourth domains are essential for STAU1 homodimerization(2). *SF3B1* mutation led to the expression of the isoform 3, which is an in-frame inclusion of part of a non-canonical exon that disrupt the NLS signal and part of the second dsRBD domain of the protein, domains essential for both activation of STAU1 and its direct binding to mRNA. Therefore, the alternative splice variants generated by *SF3B1* mutation may significantly impact both localization and function of this protein. SKIV2L presents several functional domains: a conserved motif Asp-Glu-Ala-Asp (DEAD box domain, in bright lemon green), a helicase ATP-binding domain (Hel, in green grass green), two rRNA-processing arch domains (rRNA ARCH, in light green), and a DSHCT domain (canonical and alternative isoforms). *SKIV2L* alternative splice variant includes part of an intron, which results in out-of-frame of protein that has truncated RNA binding domain with a potential to alter the function of the protein and its stability. SKIV2L is an RNA helicase part of RNA exosome degradation multiprotein complex, that can mediate the turnover of mRNAs(3), elimination of incompletely spliced RNA transcripts(3), and surveillance of protein translation(4). SKIV2L has been shown to have a direct role in unwinding telomeric DNA-RNA hybrids in vitro, preventing telomeric loss, cell cycle arrest and DNA damage response(5). Moreover, S*KIV2L* plays a part in limiting the RIG-I-like receptors antiviral response(3), limiting through unknown mechanisms the mTORC1-mediated autoinflammatory pathway(6), and in ensuring proper V(D)J recombination and Igh expression at the pro-B cell to large pre-B cell transition in B cell development (7). Therefore, SKIV2L downregulation may impact the response to R-loops resolution(5), and contribute to R-loops associated inflammation status(8). SERBP1 is and RNA binding protein that has been functionally implicated in transcriptional regulation, mRNA metabolism, specifically RNA splicing, regulation of mRNA stability, and the DNA damage response(9). However, SERBP1 structure and its RNA recognition and binding activity are poorly characterized: so far, few studies reported that SERBP1 bind preferentially to GC-rich motifs, and these motifs could include G-quadruplexes. *SEBP1* has four different isoforms, all affecting the HABP4-like domain (HABP4: intracellular hyaluronan binding protein 4). HABP is a paralog protein of SERBP1 known to bind both hyaluronic acid and regulate transcription, mRNA metabolism and DNA damage stress response(9). Beside the HABP4-like domain, SERBP1 present an R, RG,

and RRG (arginine and glycine rich) motifs: such domains has been shown crucial for RPA2 activation in the replication stress DNA damage response(10), and localization of SERBP1 at nucleoli and stress granules(11). **(D)** Integrative genomics viewer (IGV) screenshots of RNA-seq reads covering the cryptic 3'ss of *THOC1, STAU1,* and *SKIV2L* genes in *SF3B1* WT and MT Nalm-6 cells. **(E)** and **(F)** Semi-quantitative analysis of candidate 3'ASS splice variants associated with *SF3B1* MT involved in R-loop biogenesis in indicated isogenic cell lines and human B cells.

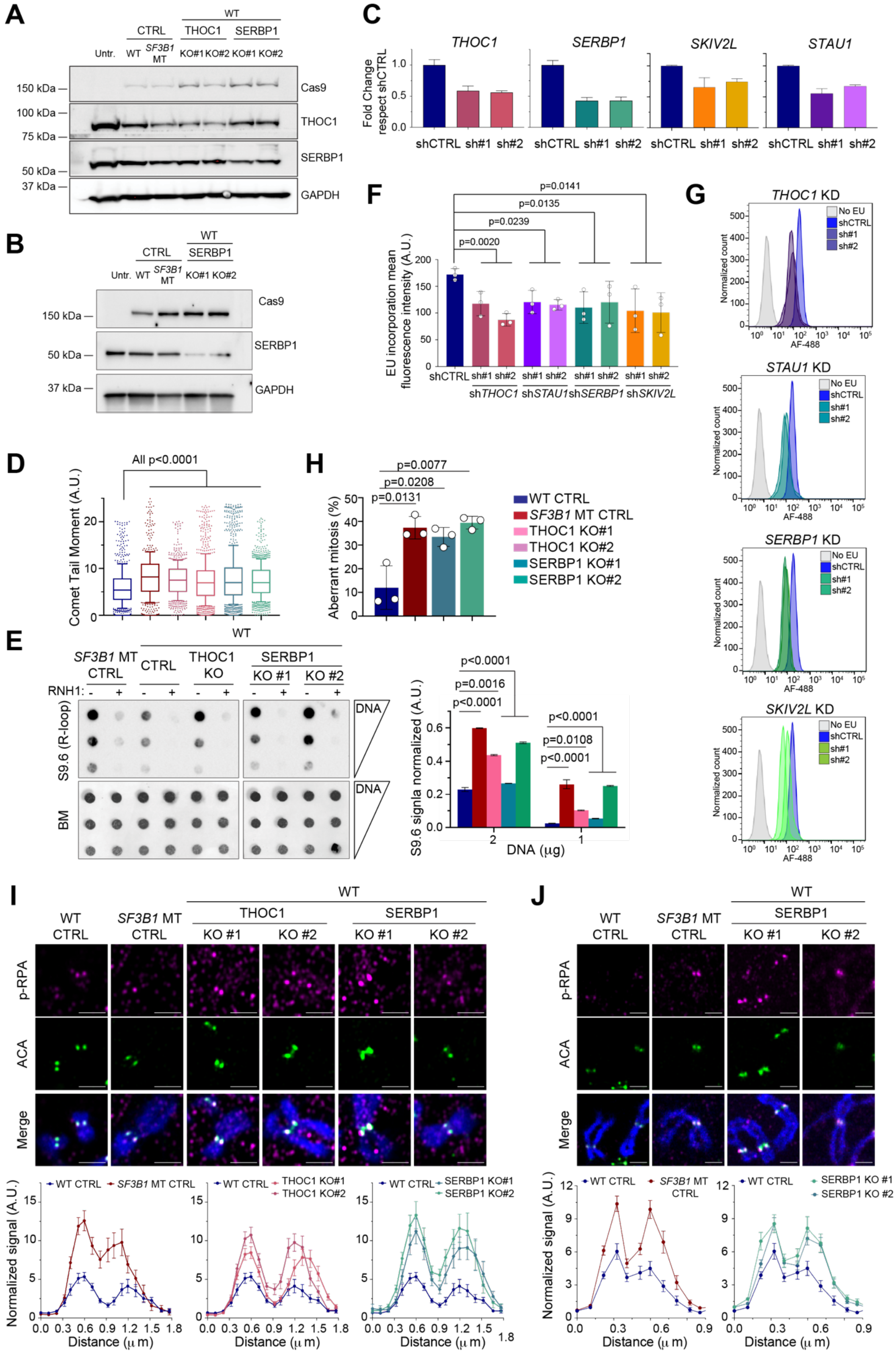

Supplemental Figure 9. ***SERBP1*** **is involved in global and centromeric R-loop metabolism.**

(**A)** THOC1 and SERBP1 protein expression assessed by immunoblot in Nalm-6 *SF3B1* WT cells with *THOC1* knockout (KO) or *SERBP1* KO**.** The KO cells were generated through CRISPR/Cas9 technology. **(B)** SERBP1 protein expression assessed by immunoblot in K562 with *SERBP1* KO. **(C)** Quantification of mRNA expression of *THOC1*, *STAU1, SERBP1*, and *SKIV2L* in HEK293T transfected with shRNAs (sh) targeting either to ROSA26 (shCTRL), or to 3'untraslated region (UTR) of *THOC1*, *STAU1*, *SERBP1*, *SKIV2L*. **(D)** Alkaline comet assay for DNA breaks detection quantification in Nalm-6 cell with either *THOC1* KO or *SERBP1* KO. The number of cells quantified: WT sgCTRL (n=892); *SF3B1* MT sgCTRL (n=693); sgTHOC1 KO#1 (n=742); sgTHOC1 KO#2 (n=1000); sgSERBP1 KO#1 (n=1199); sgSERBP1 KO#2 (n=1110). Center lines show the medians, box limits indicate the 25th and 75th percentiles, and whiskers extend to minimum and maximum values. One-way ANOVA test. **(E)** Representative dot blot images for R-loop formation (left) and quantification (right) in cells from panel (**A**). BM staining used as loading control. (**F**) and (**G**) Quantification (**F**) and Representative histograms (**G**) of EU incorporation analyzed by flow cytometry in HEK293T cells with silencing of genes showed in (**C**). Each dot represents one biological replicate. Mean ± SD was plotted Two-way ANOVA test. No EU incorporation sample was included as a negative control **(H)** Frequency of cells with aberrant mitosis expressed as % of total mitosis encountered in K562 cells: WT sgCTRL (n=29); *SF3B1* MT sgCTRL (n=53); sgSERBP1 KO#1 (n=54); sgSERBP1 KO#2 (n=42). Bar graphs represent mean ± S(D) Each dot represents a biological replicate. Two-tailed unpaired *t*-test. **(I)** and **(J)** Centromeric R-loop accumulation assayed by p-RPA immunofluorescence on chromosome spreads in Nalm-6 cells with either T*HOC1* KO or *SERBP1* KO **(I)** and in K562 Cas9 cells with or without SERBP1 KO **(J)**. In both panels, on top: representative confocal images p-RPA and centromere (ACA) immunofluorescence. Chromosome stained with DAPI (blue). Scale bar: 2µm. In both panels, on bottom: quantification of centromeric p-RPA immunofluorescence signal over background signal near centromeres. Graphs represent mean ± SEM. Nalm-6 Cas9 two-Tailed paired *t*-test: *SF3B1* WT CTRL vs. *SF3B1* MT CTRL, p-value 0.0009; *SF3B1* WT CTRL vs. THOC1 KO#1, p-value 0.0007; *SF3B1* WT CTRL vs. THOC1 KO#2, p-value 0.0002; *SF3B1* WT CTRL vs. SERBP1 KO#1, p-value <0.0001; *SF3B1* WT CTRL vs. SERBP1 KO#2, p-value <0.0001. Nalm-6 Cas9 chromosomes quantified: *SF3B1* WT sgCTRL (n=62); *SF3B1* MT sgCTRL(n=40); sgTHOC1 KO#1 (n=100); sgTHOC1 KO#2 (n=57); sgSERBP1 KO#1 (n=38); sgSERBP1 KO#2 (n=45). K562 Cas9 two-Tailed paired *t*-test: WT sgCTRL *vs*. *SF3B1* MT sgCTRL, p-value 0.0208; WT CTRL *vs*. sgSERBP1 KO#1, p-value 0.0110; WT sgCTRL *vs*. sgSERBP1 KO#2, p-value 0.0086. K562 Cas9 chromosomes quantified: *SF3B1* WT sgCTRL (n=29); *SF3B1* MT sgCTRL (n=53); sgSERBP1 KO#1 (n=54); sgSERBP1 KO#2 (n=42).

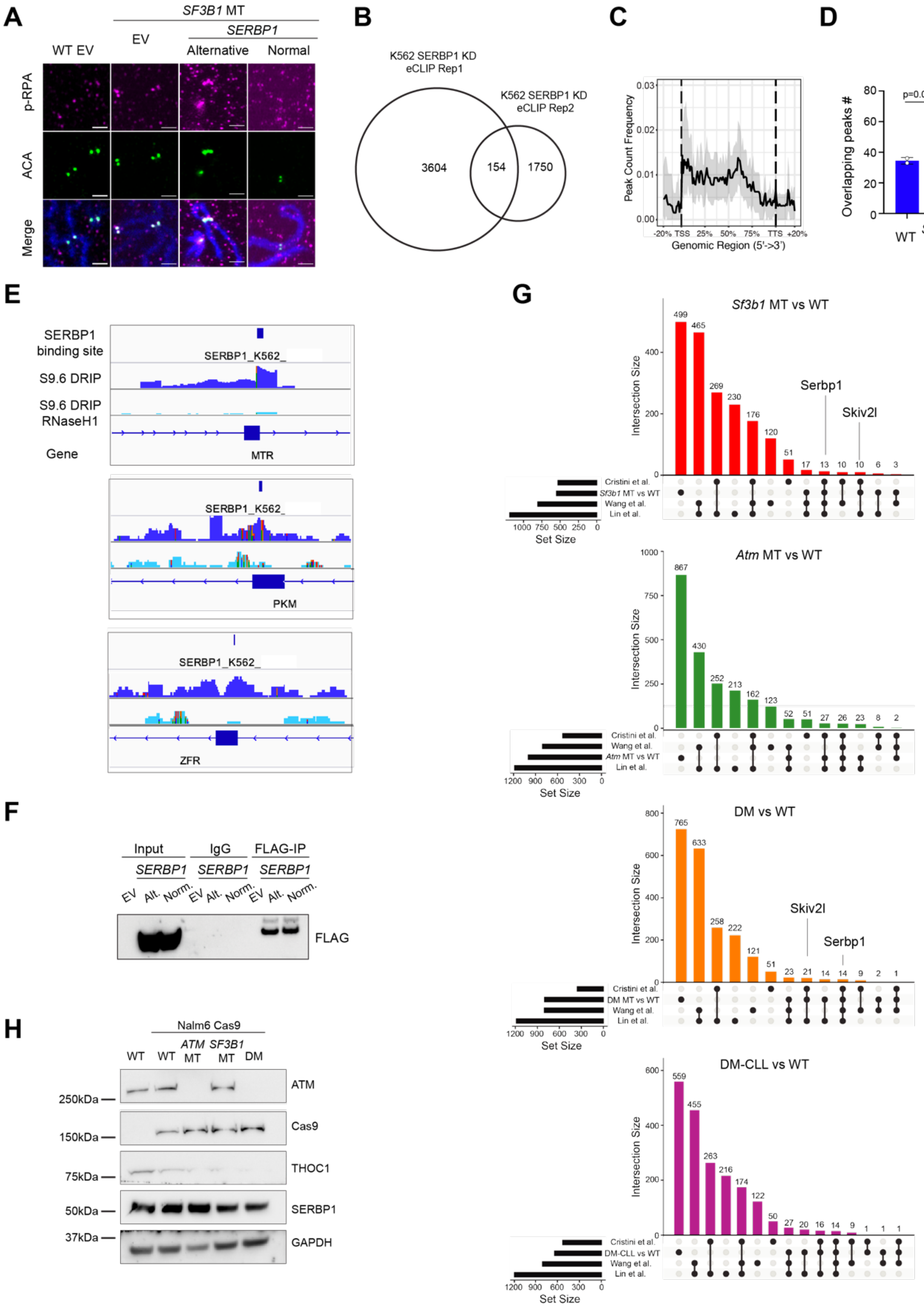

Supplemental Figure 10. ***ATM*** **deletion impacts RNA splicing in genes involved in R-loop biogenesis.** (**A**) Representative images of p-RPA (red) and centromere (ACA, green) immunofluorescence in chromosome spreads from HEK293T *SF3B1* WT and MT over-expressing either canonical or alternative SERBP1 isoform, and empty vector (EV). Chromosomes (blue) stained with

DAPI. Scale bar: 2μm. **(B)** Overlap between replicates of available SERBP1 CLIP-seq derived from K562 cells (ENCODE). **(C)** Meta analysis of SERBP1 CLIP-seq peaks distribution over transcription starting site (TSS), coding region, and transcription termination site (TTS). **(D)** Bar graph represents overlapped SERBP1 RNA binding peaks from CLIP-seq and R-loop peaks from DRIP-seq from Nalm-6 *SF3B1* WT and MT cells. **(E)** IGV screenshots of CLIP-seq peaks and R-loop peaks of indicated genes. **(F)** Representative immunoblot of FLAG immunoprecipitation performed with lysates from HEK293 overexpressing either empty vector (EV) or *SERBP1* normal (Norm) and altered (Alt) isoforms prepared for eCLIP analysis. (**G**) Upset plots show overlap of splice variants associated with different genetic lesions from murine B cells (12) with R-loop interactome and regulatome. (**H**) Representative immunoblot of ATM, THOC1, SERBP1 in NALM6 *SF3B1* WT and MT cells with or without *ATM* deletion. GAPDH used as loading control.

Table S1: Supplied as an Excel File. Primers, shRNAs, and sgRNAs oligonucleotide sequences.

Table S2: Supplied as an Excel File. *SF3B1* MT associated splice variants in HEK293T, Nalm-6, and K562 cell lines.

Supplemental References

1. Zhou Y, Zhou B, Pache L, Chang M, Khodabakhshi AH, Tanaseichuk O, et al. Metascape provides a biologist-oriented resource for the analysis of systems-level datasets. *Nature communications.* 2019;10(1):1523.
2. Almasi S, and Jasmin BJ. The multifunctional RNA-binding protein Staufen1: an emerging regulator of oncogenesis through its various roles in key cellular events. *Cellular and Molecular Life Sciences.* 2021;78(23):7145-60.
3. Eckard SC, Rice GI, Fabre A, Badens C, Gray EE, Hartley JL, et al. The SKIV2L RNA exosome limits activation of the RIG-I-like receptors. *Nature Immunology.* 2014;15(9):839-45.
4. Tuck AC, Rankova A, Arpat AB, Liechti LA, Hess D, Iesmantavicius V, et al. Mammalian RNA Decay Pathways Are Highly Specialized and Widely Linked to Translation. *Molecular Cell.* 2020;77(6):1222-36.e13.
5. Herrera-Moyano E, Porreca RM, Ranjha L, Skourti E, Gonzalez-Franco R, Sun Y, et al. The human SKI complex prevents DNA-RNA hybrid-associated telomere instability. *bioRxiv.* 2021:2020.05.20.107144.
6. Yang K, Han J, Asada M, Gill JG, Park JY, Sathe MN, et al. Cytoplasmic RNA quality control failure engages mTORC1-mediated autoinflammatory disease. *J Clin Invest.* 2022;132(2).
7. Yang K, Han J, Gill JG, Park JY, Sathe MN, Gattineni J, et al. The mammalian SKIV2L RNA exosome is essential for early B cell development. *Science Immunology.* 2022;7(72):eabn2888.
8. Chatzidoukaki O, Stratigi K, Goulielmaki E, Niotis G, Akalestou-Clocher A, Gkirtzimanaki K, et al. R-loops trigger the release of cytoplasmic ssDNAs leading to chronic inflammation upon DNA damage. *Sci Adv.* 2021;7(47):eabj5769.
9. Colleti C, Melo-Hanchuk TD, da Silva FRM, Saito Â, and Kobarg J. Complex interactomes and post-translational modifications of the regulatory proteins HABP4 and SERBP1 suggest pleiotropic cellular functions. *World J Biol Chem.* 2019;10(3):44-64.
10. Ahn JW, Kim S, Na W, Baek SJ, Kim JH, Min K, et al. SERBP1 affects homologous recombination-mediated DNA repair by regulation of CtIP translation during S phase. *Nucleic Acids Res.* 2015;43(13):6321-33.
11. Lee Y-J, Wei H-M, Chen L-Y, and Li C. Localization of SERBP1 in stress granules and nucleoli. *The FEBS Journal.* 2014;281(1):352-64.
12. Wang L, Brooks AN, Fan J, Wan Y, Gambe R, Li S, et al. Transcriptomic Characterization of SF3B1 Mutation Reveals Its Pleiotropic Effects in Chronic Lymphocytic Leukemia. *Cancer Cell.* 2016;30(5):750-63.
